# Development of novel natto using legumes produced in Europe

**DOI:** 10.1016/j.heliyon.2024.e26849

**Published:** 2024-02-29

**Authors:** Rebecca Rocchi, Jasper Zwinkels, Merit Kooijman, Alberto Garre, Eddy J. Smid

**Affiliations:** a Food Microbiology, Wageningen University and Research, Wageningen, the Netherlands; b Department of Agricultural Engineering & Institute of Plant Biotechnology, Universidad Politécnica de Cartagena, Spain

**Keywords:** Fermentation, *Bacillus subtilis*, Soy, Lentils, Chickpeas, Green peas, Vitamin K_2_, Thiamine, Pyrazines

## Abstract

Natto is a traditional Japanese fermented product consisting of cooked soybeans fermented with *Bacillus subtilis* var. natto. We assessed three different *B. subtilis* strains and investigated their impact on product quality aspects, such as microbial quality, textural quality (poly-γ-glutamate strand formation), free amino acids (FAA), and volatile organic compounds (VOCs), but also the vitamin K_1_, K_2_ and B_1_ content, and presence of nattokinase. Using Bayesian contrast analysis, we conclude that the quality attributes were influenced by both the substrate and strain used, without significant differences in bacterial growth between strain or substrate. Overall, all the tested European legumes, except for brown beans, are adequate substrates to produce natto, with comparable or higher qualities compared to the traditional soy. Out of all the tested legumes, red lentils were the most optimal fermentation substrate. They were fermented most consistently, with high concentrations of vitamin K_2_, VOCs, FAA.

## Highlights

• The production of natto using alternative legumes to soy is possible
• Red lentils, green peas, chickpeas, and lupins outperformed soybeans as substrate
• Natto made with green peas and inoculated with strain 2.8 had highest VOCs levels
• Fermented red lentils contained highest vitamin K_2_ and B_1_ content

## 1 Introduction

Itobiki-natto, also known as natto, is a traditional Japanese fermented food. It is produced by washing, soaking, and cooking soybeans before inoculation with *Bacillus subtilis* var. natto. The fermentation is followed by a maturation step before the product is ready for consumption. Fermentation is the most critical part of the natto production process, since it has a great impact on the final product characteristics such as texture, appearance, flavour, and nutritional value [1].

Natto has a typical sticky-slimy texture given by the γ-polyglutamic acid (PGA) strands produced by *B. subtilis* during fermentation and maturation [2]. These strands are a biopolymer constituted by variable proportions of D-form and L-form glutamic acid linked by γ-glutamyl bonds. In addition, soy is a rich source of proteins, and during fermentation the proteolytic activity of *B. subtilis* releases ammonia and free amino acids, which contribute to the smell and taste of the product, whilst increasing the overall digestibility [3]. Free amino acids (FAA) contribute directly to taste and flavour perception of natto since most amino acids are either sweet, bitter, or umami in taste. Furthermore, FAA are precursors of other metabolites contributing to the quality of natto. Glutamic acid for instance is the building block of γ-PGA, giving natto its slimy texture [4]. Furthermore, they are also precursors of many secondary metabolites (SM) characterizing the aroma profile of natto, such as pyrazines and ketones [[5], [6], [7], [8], [9]]. Ammonia is the major SM contributing to natto aroma, followed by 2,5-dimethylpyrazine and acetoin, arising from conversions catalysed by *B. subtilis* [7,10,11]. Pyrazines and especially 2,5-dimethylpyrazine are potent molecules with a high flavour dilution and deliver a nutty or baked nut-like smell [8]. Acetoin itself has a high odour threshold but is easily oxidized to diacetyl (2,3-butanedione), which has a buttery aroma. Minor common contributions to natto aroma are beany flavours, such as benzaldehyde and 2-pentylfuran originating from legumes [8,12].

An important vitamin present in natto is thiamine (vitamin B_1_), this vitamin is both of plant and microbial origin. *B. subtilis* can synthesize thiamine *de novo* as well as salvage it from the environment [13]. Thiamine vitamers include the free unphosphorylated from, as well as various phosphorylated ones. The free unphosphorylated thiamine is a precursor of flavour compounds, such as 2-methyl-3-furanthiol, 2-methyl-3-methyldithiofuran, and bis(2-methyl-3-furyl)disulphide, that derive from the thermal degradation that occurs during cooking, these compounds have a strong meaty and roasted aroma [14,15]. Whereas its phosphorylated form, thiamine pyrophosphate is biologically active as a co-factor of many enzymes with an important role in human metabolic functions, indeed a severe deficiency causes beriberi syndrome, that can lead to death [16].

An increase in natto consumption made with legumes produced in Europe is not only favourable for a sustainability stance, but also for the health-promoting effects that have been attributed to natto consumption. Examples are anti-hypertensive [17] anti-thrombotic [18], and anti-diabetic [19] activities as well as reduction in cardiovascular mortality [20]. These health benefits are attributed to bioactive compounds in natto such as vitamin K_2_, and nattokinase [[21], [22], [23]].

In natto we find two variants vitamin K: phylloquinone (vitamin K_1_), and menaquinone (vitamin K_2_). Both are important in human health, since they contribute to many biological activities related to haemostasis, calcium, and bone metabolism [21,24,25]. Phylloquinone is of plant origin, therefore is already present in soy, while menaquinone - specifically MK-7 - is produced by *B. subtilis* during fermentation. Natto has significantly higher amounts of menaquinone compared to most plant-based foods, and it is one of the richest sources of this vitamin [26]. Another compound present in natto that is produced via fermentation is an enzyme of the subtilisin family known as nattokinase. Nattokinase is a trypsin-like serine protease made up of a single polypeptide chain composed of 275 amino acids [27,28]. It is stable under acidic conditions and can enter the blood stream intact by intestinal absorption through the epithelial cells or through tight junctions [29,30]. In animal and human trials nattokinase has shown potent fibrinolytic [28,30], anti-hypertensive [[31], [32], [33]], anti-atherosclerotic [34,35], lipid-lowering [34,36], anti-platelet/anti-coagulant [37] and neuroprotective actions [38,39]. Fibrinolytic activity of nattokinase has been observed to degrade blood clots *in vivo* and *in vitro* and works through direct degradation of fibrin to fibrin degradation products, or through indirectly activating plasminogen to plasmin, which in turn degrades fibrin [27,28]. These properties make natto and nattokinase relevant in the prevention and treatment of cardiovascular diseases (CVD) [40].

Soy, the traditional substrate for natto, is a sub-optimal crop to promote for large consumption in Europe, since it is an allergen, it is not native to Europe, and the European Union is heavily reliant on its import. Therefore, natto production with European legumes can increase food security and reduce reliance on import. For this reason, to further improve the sustainability of natto as an alternative protein source, it is relevant to study the use of legumes cultivated in Europe as alternative fermentation substrates [41,42]. In this study we selected five legumes that are cultivated in Europe: brown beans, green peas, and lupin, which are commonly produced in Central and Northern Europe; and red lentils, and chickpea, which are mostly produced in Southern Europe [43,44]. The rising interest in fermented products, and natto’s nutritional profile, show potential for further development of the European market of natto. Therefore, this study aimed to develop a novel natto product made with the previously mentioned European legumes. To study the impact of the choice of strain on the final product we assessed three different *B. subtilis* strains on these substrates, and the natto obtained were compared to those produced using soy as the reference substrate. The comparison was done on the microbial growth and alkalinization, the production of γ-polyglutamic acid strands, the release of free amino acids, the content of vitamin B_1_, K_1_, and K_2_, and the amount of nattokinase produced.

## 2 Materials and methods

### 2.1 Legumes and bacterial strains

Brown beans (*Phaseolus vulgaris*), chickpeas (*Cicer arietinum)*, green peas (*Pisum Sativum)*, split dehulled lupins (*Lupinus albus)*, red lentils (*Lens culinaris)* and soybeans (*Glycine max)* were purchased from DO-IT Organic (Barneveld, Netherlands). They were stored sealed at room temperature until use. Three *Bacillus subtilis* strains were used for this study, two wild-type strains isolated from natto, namely 1.8 and 2.8, and DSM 1092 strain, purchased from DSMZ (German Collection of Microorganisms and Cell Cultures GmbH, Braunschweig, Germany). Strain 1.8 was isolated from natto from Takano Foods (Japan), and 2.8 was isolated from natto by NATTODAN (The Netherlands). In this paper we refer to strain DSM 1092 as 92. Strains were stored at −80 °C in 30% glycerol.

### 2.2 Inoculum preparation

A cell suspension of *B. subtilis* var. natto was prepared from a glycerol stock on nutrient agar plates (Thermo Fisher, Manchester, UK) and incubated at 30 °C for 15 h, and afterwards at 4 °C for 48 h. For each strain, one colony was taken from that plate and put into a 50 mL Erlenmeyer with 25 mL nutrient broth (nutrient agar composition omitted of agar) and incubated at 30 °C at 180 rpm for about 22 h 20 mL of overnight culture were washed twice in 20 mL of PBS 2 ml of PBS. This cell suspension had a concentration of about 8 log_10_ CFU/mL.

### 2.3 Natto production

Natto was prepared according to the method of Wei et al. [45], with some adaptions. In brief, legumes (except green peas and red lentils) were washed and soaked for 16 h at 25 °C with bean:water ratio 1:3. Legumes were cooked in a Presto 23-quart pressure canner and cooker (National Presto Industries Inc., Eau Claire, WI, USA) at 41 kPa (kPa) at 110 °C. The soybeans, lupin and chickpeas were cooked for 30 min, the brown beans and green peas were cooked for 22 min, and the red lentils for 15 min. Legumes were dried in open air on a metal mesh (cooling tray) for 2.5 h. 200 g of legume was manually mixed in a bag with 0.2 mL of inoculum (with about 8 log_10_ CFU/mL). Each sample was put in a sterile fermentation container of 14 × 9 × 3.5 cm with holes of 1 mm diameter 3 cm apart. Samples were fermented at 37 °C, static, under constant conditions for 24 h, followed by a maturation step at 4 °C for 24 h.

### 2.4 Total viable count

To estimate the viable plate count of *B. subtilis* in natto, 10 g of natto together with 90 mL PBS were added to a stomacher bag and homogenised in a stomacher at 230 rpm for 1 min. The samples were appropriately diluted in PBS and a 0.1 mL was spread plated on a nutrient agar plate and incubated at 37 °C for about 16 h. Colonies present on the overnight plate were counted for total viable count enumeration.

### 2.5 pH measurement

The pH of the samples was measured before and after fermentation according to the following procedure. Ten grams of cooked legumes or natto were weighed in a 50 mlLGreiner tube with 20 mL demineralised water. Then the samples were homogenised using an ULTRA-TURRAX® T 25 (IKA-Werke GmbH, Germany) for about 30 s at 16.000 rpm, until a thick slurry was formed, the pH was measured using a pH meter (MeterLab PHM240, Denmark).

### 2.6 Polyglutamic acid analysis

To analyse γ-polyglutamic acid formation, the samples were vigorously mixed with a sterile spoon for 10 s and pictures were taken of all the sample. The pictures were visually examined and to each sample a value between 0 and 3 was assigned. A value of 0 indicates no formation of strands, a value of 3 indicates that a high amount of thick strands was formed.

### 2.7 Sample preparation

To prepare the natto samples for analysis of free amino acids, thiamine, and vitamin K_1_ and K_2_ and nattokinase, 10 g of each sample was frozen in liquid nitrogen, after evaporation of nitrogen the frozen samples were transferred into a coffee grinder (Krups F203, Solingen, Germany**)** and ground up for 20 s obtaining a fine powder. The homogenised samples were by −20 °C until further analysis.

### 2.8 Thiamine extraction and determination

An aliquot of the ground-up frozen natto, 2.5 g, was placed in a tube and diluted 10 times with 22.5 mL 0.1 M HCl, and extracted for 30 min in a water bath at 95 °C. Afterwards, 1 mL of the extract was diluted with 9 mL 0.1 M HCl and filtered using a 0.2-μm filter and stored at −20 °C before derivatization and HPLC analysis as described by Rocchi and co-workers [46], with the only modification of the pH of the mobile phase A, which was set to 7.

### 2.9 Vitamin K_1_ and K_2_ extraction and determination

The method of Tsukamoto, Ichise and co-workers [23] was adapted as described below for the extraction of vitamin K_1_ and K_2_. An aliquote of 2.5 g of ground-up frozen natto was placed in a tube and diluted 10 times with 22.5 mL of demineralised water. As an internal standard 200 μL of 5.2 mg/L MK-4 was added to each sample, along with 5 mL of isopropanol. The solution was mixed for 10 min at 220 rpm, and 6 mL hexane (EMSURE®, Sigma-Aldrich) was added, followed by a second mixing step for 10 min at 220 rpm. The phase separation was accelerated with centrifugation for 10 min at 3000 g. The upper phase was collected and put in a glass bottle. This extraction with 6 mL hexane was repeated another time, and the second upper phase was added to the first. Finally, the hexane was evaporated using a nitrogen flow for about 45 min. The glass vials containing the extracted K_2_ were rinsed with 0.5 mL of isopropanol (EMSURE®, Sigma-Aldrich). The dissolved K_2_ was put in a dark glass vial and stored at −20 °C until analysis. The samples were analysed using liquid chromatography coupled with mass spectrometry (LC-MS). Vitamin K_2_ determination was done as described by Liu and co-workers [47], with the following modifications. The volume of injected sample was 35 μL, the mobile phase A was demineralised water and mobile phase B was acetonitrile (EMSURE®, Sigma-Aldrich). Standard solution of MK-4 (Sigma-Aldrich), MK-7 (Sigma-Aldrich), and vitamin K_1_ (Sigma-Aldrich) in the concentration range from 1 ng/mL to 3 μg/mL were analysed to obtain the calibration curves.

### 2.10 Determination fibrinolytic activity

Nattokinase concentration was determined as a function of fibrinolytic activity in natto, unfermented legumes, and overnight cultures of *B. subtilis* strains on nutrient broth. Sterile nutrient broth Plasminogen (Sigma-Aldrich, St. Louis, Missouri, USA) and commercial nattokinase (Brand: Doctor’s Best Inc; obtained from Superfoodsonline.nl, Groningen, The Netherlands; manufactured in Tustin, California, USA; 10 and 100 FU/mL; additional ingredients: maltodextrin, Hypromellose, magnesium stearate) were used as negative and positive control, respectively. Fibrin gels were made in 12-wells plate (Ø23 mm) by successive addition of 1 mL fibrinogen (plasminogen depleted, from human plasma; Sigma-Aldrich; 5 mg/mL), 0.1 mL Tris-HCl buffer (50 mM, pH 7.0), 0.1 mL thrombin (Sigma-Aldrich; 20 U/mL) and 0.78 mL agarose solution (1%). Plates were left to solidify. Natto and legume samples were prepared by suspending 1 g of sample in 5 mL Tris-HCl buffer. Samples were stored overnight at 4 °C and incubated 30 min at 37 °C in shaking water bath for 30 min, directly before measurement. Overnight cultures were grown on nutrient broth for 24 h at 37 °C at 160 rpm and subsequently OD was measured. All samples were centrifuged (6400 g, 5 min) and 3 μL of the supernatant was applied in the centre of the gel. Plates were incubated overnight at 37 °C and diameter of clear zone was measured. Results. Commercial nattokinase (2000 fibrinolytic units/capsule) was used as a standard.

### 2.11 Free amino acid content determination

Free amino acid profile was determined by Ultra-High Pressure Liquid Chromatography (UPLC) using AccQ-Tag Ultra Derivatization Kit (Waters, Millford, MA, USA). The procedure of Scott et al. [48] was followed with some adaptions. The adaption being that 1 g of ground-up frozen natto was suspended in 9 g of demi water.

### 2.12 Volatile organic compound analysis

Volatile organic compounds (VOC) present in natto were analysed using Gas chromatography-mass spectrometry (GC-MS) following the procedure of Scott et al. [48]. Only adaption was that samples were prepared by putting about 1.1 g of each in 5 mL GC-MS glass vials, capped and stored at −20 °C until analysis. The peak identification was done by comparing the mass spectra with the profiles in the NIST (National Institute of Standards and Technology) main library, and the peak areas were determined via the MS quantitation peak, considering the highest *m*/*z* peak for each compound.

### 2.13 Statistical analysis and graphical representation

The effect of product and strain on the quality attributes measured was evaluated by Bayesian linear contrast analysis [49].

For the contrast analysis, the strain and product effects on each quality attribute (y) was described as a multilevel linear model, as shown in Equation (1). Accordingly, the quality attribute follows a normal distribution with expected value, ***μ***, and unknown variance ***σ***.

(1) y ∼ Normal(***μ***, ***σ***)

The strain and product affects are defined as linear perturbations on ***μ***. As shown in Equation (2), the product effect is quantified by the deviation, ***ε***_p_, with respect to the grand mean a_0_. Then, the strain-effect for each product is modelled as a nested perturbation ***ε***_s|p_. Both perturbations are assumed to follow a normal distribution with mean zero and unknown variances ***σ***p and ***σ***s.

(2) ***μ*** ← a_0_ + ***ε***_p_ + ***ε***_s|p_

***ε***_p_ ∼ Normal(0, ***σ***_p_)

***ε***_s|p_ ∼ Normal (0, ***σ***_s_).

All the calculations were done in R version 4.2.1 [R Core Team (2022)].The models were fitted using the no-U-turn Hamiltonian Monte Carlo sampler included in Stan [50] using the brms package [51]. The convergence of the model fit was evaluated by visualising the trace plots of the Markov chains and, as well as ensuring the R-hat index was lower than 1.01, resulting in four independent chains with 6,000 iterations and 3,000 warmup iterations.

The contrast for each product, i, (C_p[i]_) was defined as the difference with respect to the grand mean as shown in Equation (3). It was calculated directly from the Monte Carlo iterations, determining the significance of the product effect based on the quantiles of the posterior distribution.

(3) C_p[i]_ = a_0_ - ***ε***_p[i]_

The contrast for each strain, j, conditional to each product, i, (C_s[j]|p[i]_) was defined as the difference with respect to control (uninoculated) conditions as shown in Equation (4). Again, the posterior of the contrast was calculated directly from the Monte Carlo iterations.

(4) C_s[j]|p[i]_= (a_0_ + ***ε***_p[i]_+ ***ε***_s[control]|p[i]_) - (a_0_ + ***ε***_p[i]_+ ***ε***_s[j]|p[i]_)

Data were stored using Excel software v.16.0 (Microsoft Corporation, Redmond, WA, USA), data analysis and figures were done using R version 4.2.1.

HPLC-MS data were processed using MassLynx software (Waters). The peak areas of the samples were adjusted based on the extraction yield on the MK-4 internal standard. HPLC-Fluorescence chromatogram data was processed using Chromperfect v. 6.0.18 (Denville, NJ, USA). Data generated by GC-MS were analysed in Chromeleon 7.3.1 (Thermo Scientific, MA, USA).

## 3 Results

### 3.1 Microbial activity

**Fig. 1:**
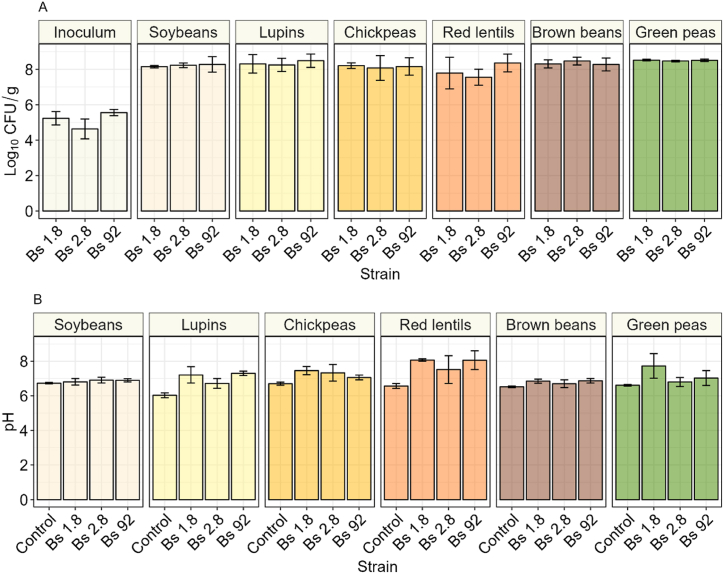
**A** Total viable count of inoculum and fermented natto samples after fermentation and maturation, on nutrient agar. **B** pH of control and fermented natto samples after fermentation and maturation on nutrient agar. The error bar represents the standard deviation of the means of three biological replicates.

The growth of *B. subtilis* on the different legumes affects the pH and the development of the typical texture of natto, which are indicators of a successful fermentation. Samples inoculated with strain 2.8 had on average 4.6 log_10_ CFU/g at the onset of fermentation, while strain 1.8 and 92 had respectively 5.2 and 5.6 log_10_ CFU/g (Fig. 1A). Bacterial growth occurred in all the samples, with no significant difference between different strains or substrates at the end of the maturation process. The highest viable bacterial counts were found in all the soybean samples and in chickpea natto inoculated with strain 92, with about 8.5 log_10_ CFU/g. The lowest cell counts were found on lupin inoculated with strain 2.8, where the average microbial concentration was 7.6 log_10_ CFU/g. In general, the choice of substrate had more influence on the final bacterial cell count compared to the choice of strain, but overall, the total microbial concentration was comparable across all the natto products.

The unfermented legume with lowest pH was lupin, with a value of 6.0, while the other legumes had an initial pH between 6.5 for brown beans and 6.7 for soybeans (Fig. 1B). After maturation, there was no significant difference in pH between fermented and unfermented substrates in both soybeans and brown beans. In these two substrates the pH reached a value of 6.9 with strain 2.8 and 92 in soybeans, and with strain 1.8 and 92 in brown beans. Green peas 1.8 had a significantly higher pH, compared to the control, while green peas 2.8 and 92 did not. In chickpea natto, the only sample that did not increase significantly compared to the control was strain 92. All the fermented lupin and red lentils samples were significantly higher compared to the control condition. Overall, the highest alkalinization was observed in red lentils, with an average pH of 8.1 for both strain 1.8 and 92, compared to the control that had an initial pH of 6.5 in this case both the choice of strain and substrate impacted the outcome of the alkalinization process.

Overall, the natto with the highest amount of strands formed was chickpeas, and the lowest were brown beans and red lentils (Table 1 and Supplementary material 25). The highest γ-polyglutamate (PGA) producing strain was strain 2.8, while the lowest was strain 92, which produced no strands at all in brown beans and red lentils, and few strands in all the other substrates, except for chickpeas.

**Table 1:** Table indicating the average value (n = 3) of the string formed in the natto samples, assessed via visual observation.

Strain	Soybeans	Lupins	Chickpeas	Red lentils	Brown beans	Green peas	Strain average
**Bs 1.8**	2	2	2	1	2	1	1.7
**Bs 2.8**	2	2	3	2	1	2	2.0
**Bs 92**	1	1	2	0	0	1	0.8
**Legume average**	1.7	1.7	2.3	1.0	1.0	1.3	

### 3.2 Vitamin B_1_ content

**Fig. 2:**
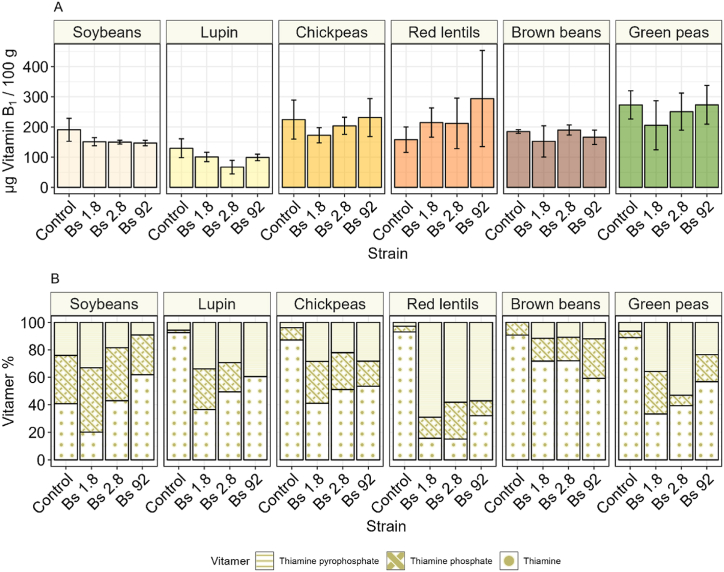
**A** Total thiamine content of control and fermented natto samples after fermentation and maturation. **B** Vitamin B_1_ vitamer distribution percentage of control and fermented natto samples. The error bar represents the standard deviation of the means of three biological replicates.

Thiamine can be synthetized *de novo* by plants and bacteria, and it is not only important as an essential nutrient in the human diet, but also as it is an important precursor of meat-like and umami-tasting molecules [52]. This vitamin was detected in all the unfermented samples (Fig. 2A), and its concentration did not significantly change for any of the legumes during fermentation and after maturation. Overall, lupin had a lower concentration of thiamine compared to the other substrates assessed in this study. The choice of *B. subtilis* var. natto strain did not impact the overall presence of thiamine, because the fermentation did not significantly change the amount of thiamine detected. Green peas had the highest amount of thiamine found in the unfermented substrates, on average 273 μg/100 g, and lupin had the lowest, with 129 μg/100 g.

While the total thiamine concentration did not change in all the fermented substrates, the distribution of the vitamers did. The relative contribution of unphosphorylated thiamine decreases during the fermentation while the relative contribution of phosphorylated and diphosphorylated increased (Fig. 2B), except for soy fermented with strain 2.8 and 92. Especially in red lentils, the free form of thiamine went from representing over 93% of the total thiamine to 69%, 58%, and 57% in samples 1.8, 2.8 and 92. Soy fermented with strain 92 was the only substrate for which there was an increase in proportion of thiamine, where free thiamine went from 40 to 61%. Instead, for soy fermented with strain 1.8, thiamine decreased, while thiamine phosphate and pyrophosphate increased. Soy fermented with strain 2.8 showed virtually unchanged proportions of the different vitamers. This change in the different vitamers pool before and after fermentation is a sign of microbial activity, since only thiamine pyrophosphate is biologically active, and the heat treatment of the substrate is expected to inactivate all the plant derived enzymes.

### 3.3 Vitamin K_1_ and K_2_ content

**Fig. 3:**
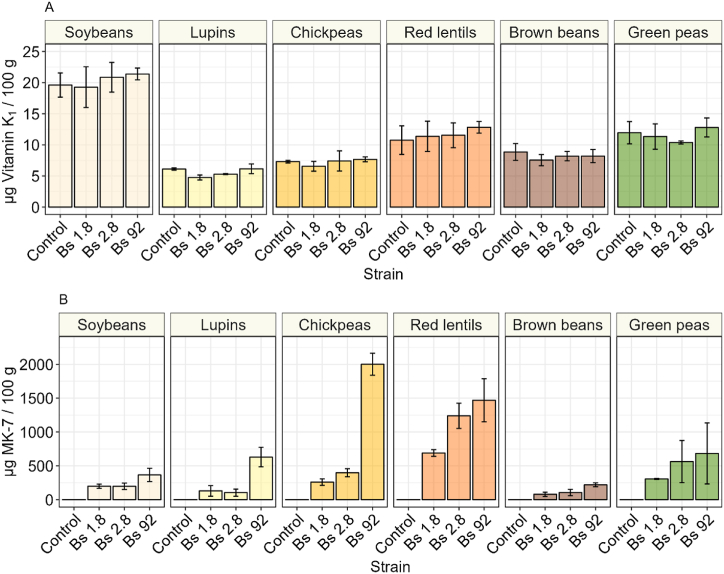
**A** Vitamin K_1_ content in fermented natto samples, after fermentation and maturation, on nutrient agar. **B** Vitamin K_2_ content of natto samples after fermentation and maturation. The error bars represent the standard deviation of the means of three biological replicates.

Vitamin K_1_ and K_2_ are known to be present in natto and have a well-established positive influence on human health. Each one of these vitamers is specific to the organism that produces it. Phylloquinone (vitamin K_1_) is exclusively of plant origin, vitamin K_2_ can be both of animal and bacterial origin. Menaquinone 7 (MK-7) is produced during fermentation by *B. subtilis,* and it is exclusively of microbial origin. In this study we used the animal derived menaquinone 4 (MK-4), as internal standard to determine the efficiency of extraction of MK-7 and vitamin K_1_. Phylloquinone was found in all the fermented and unfermented samples, and its concentration was unaffected by fermentation and maturation (Fig. 3A). Soy had significantly higher concentration of phylloquinone compared to all the other substrates, with on average 19.6 μg/100 g. The lowest concentration of phylloquinone was found in lupin, with 6.1 μg/100 g on average, this amount was significantly lower compared to all other substrates. In chickpeas and brown beans, we found respectively 7.3 and 8.9 μg/100 g of phylloquinone, while in green peas and red lentils had 12 μg/100 g and 10.8 μg/100 g of phylloquinone, respectively.

As expected, no MK-7 was found in any of the unfermented substrates (Fig. 3B). During fermentation and maturation MK-7 increased significantly in all the samples. Strain 92 consistently produced more MK-7 on average compared to all the other strains. Whereas substrate-wise, red lentils had on average higher MK-7 concentrations, with respectively 690, 1283, and 1647 μg/100 g for strain 1.8, 2.8, and 92. The highest amount of MK-7 was found in chickpeas fermented with strain 92, with on average 2002 μg/100 g. The lowest average amount of MK-7 was produced in brown beans, inoculated with strain 1.8, in which only 78.9 μg/100 g was found. Overall, both the choice of strain and substrate impacted the production of vitamin K_2_ and strain 92 consistently yielded more menaquinone.

### 3.4 Fibrinolytic activity

**Fig. 4:**
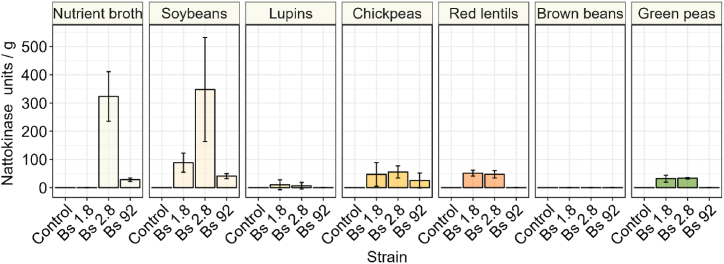
Nattokinase content of nutrient broth, control, and fermentation natto samples after fermentation and maturation. The error bars represent the standard deviation for the means of three biological replicates.

In this study we detected and quantified fibrinolytic activity in the different natto samples, and supernatants of overnight cultures by monitoring the diameter of the clearance zone in fibrin plates upon the addition of sample extracts (Fig. 4). Sterile nutrient broth and commercially available nattokinase were used as negative and positive control, respectively, and for calibration. The fibrinolytic activity detected varied depending on the strain and substrate used for natto production. Overall, nattokinase production was influenced more by strain than substrate (based on Bayesian contrast analysis). No clear zone was observed in unfermented legumes. Fibrinolytic activity was only observed in fermented natto samples, with a clear zone from 0.3 to 11.0 mm, corresponding to 5 to 350 nattokinase units/g. Only fermented brown beans did not show any fibrinolytic activity. The lowest nattokinase producing strain was strain 92. The highest nattokinase producing legume was soy, on average. Commercial nattokinase supplement diluted to match 10, 100, 500, and 1000 nattokinase units produced respectively 5.5, 15.0, 19.0, and 23 mm clear zones. In soy, fermented with strain 1.8 and 2.8 88 units/g and 347 units/g were detected, respectively. This was the only instance that the same strain produced more nattokinase in a legume, compared to nutrient broth. The lowest detectible nattokinase activity (7 units/g) was observed in lupin natto fermented with strain 2.8.

### 3.5 Free amino acids and ammonia content

During fermentation, proteolytic activity of *B. subtilis* leads to the release of free amino acids (FAAs) [53]. The pool-size of the free amino acids depends on rate of protein hydrolysis, and the rate of amino acid update by the fermenting microorganisms. The pool-size of the FAAs is a predictor of natto quality, as well as an indicator of successful fermentation, since the FAAs are precursors of many volatile organic compounds, and other relevant metabolites. For instance, glutamic acid is the precursor of γ-PGA. FAA content in unfermented legumes was relatively low, around 0.8 ± 0.2 mmol/100 g, but fermentation resulted in an increase in free amino acid pools. Brown beans were the exception. In this fermentation the total free amino acids were reduced. Unfermented brown beans contained 0.7 ± 0.05 mmol/100 g, while brown beans fermented with strain 1.8 and strain 2.8 contained 0.24 ± 0.05 and 0.32 ± 0.04 mmol/100 g, respectively. The highest increase in FAA occurred in red lentils, for which strain 1.8 and strain 2.8 resulted in approximately a 10-fold increase, going from 0.9 mmol/100 g to between 10.5 and 10.7 mmol/100 g, respectively. Strain 92 had a lower, but still substantial increase of up to 6 mmol/100 g. Apart from brown beans, the lowest increase in FAA occurred in natto from soybeans in which the amount of FAA increased 4-fold at best in strain 2.8, from 0.5 mmol/100 g to 0.2 mmol/100 g. In general, after maturation green peas, lupins, and red lentils had significantly higher free amino acid pools as compared to the control. Whereas for the other legumes this increase was not significantly higher, except for chickpeas fermented with strain 2.8. Overall, the production of free amino acids was impacted the most by the choice of legume, rather than the strain that was used for fermentation. Furthermore, fermentations with strain 1.8, 2.8 and 92, respectively, resulted in similar FAA profiles (Fig. 6). Within samples, production of free glutamic acid, leucine and lysine was particularly high (Fig. 6). This trend in free amino acid profile was observed in all strains for lupins, chickpeas, red lentils, and green peas.

**Fig. 5:**
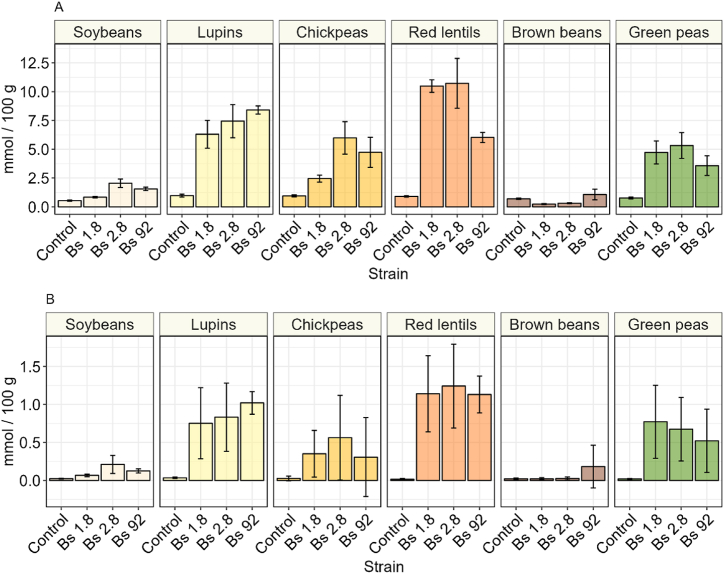
**A** Total concentration of free amino acids in control and fermented natto samples. **B** Total ammonia concentration in control and fermented natto samples, after fermentation and maturation. The error bars represent the standard deviation of the means of three biological replicate.

Ammonia concentration was highest in red lentils fermented with strain 2.8 and strain 1.8, with respectively 1.1 ± 0.5 and 1.2 ± 0.6 mmol/100 g (Fig. 5). Following fermented red lentils, ammonia concentration was highest in descending order in fermented lupins, green peas, chickpeas, soy, and brown beans. Ammonia production positively correlated with the glutamate and total free amino acid content. These legumes similarly contained highest concentrations of ammonia of all substrates (Fig. 5).

**Fig. 6:**
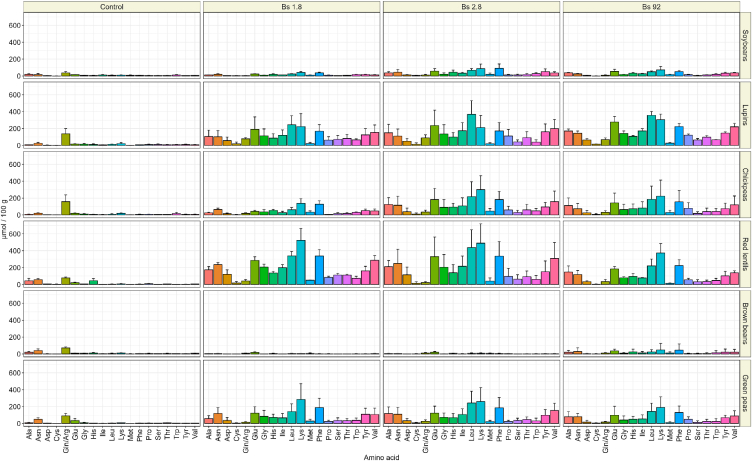
Full amino acid profile of control and fermented natto samples. Each different colour represents a single amino acids, except for glutamine and arginine which are not detected non -specifically with the HPLC method. The error bar represents the standard deviation of the means of three biological replicates.

### 3.6 Volatile aroma profile

**Fig. 7:**
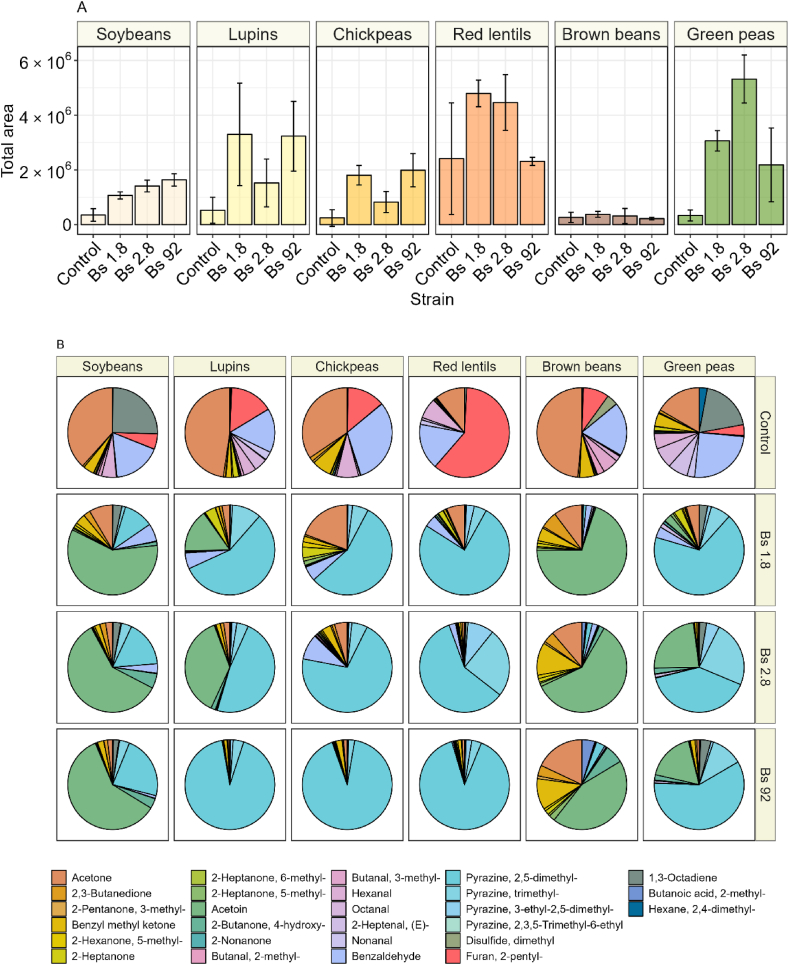
**A** Total concentration of VOC of control and fermented natto samples after fermentation and maturation. The error bar represents the standard deviation of the means of three biological replicates. **B** Distribution of VOC of control and fermented natto samples, each colour indicates a different compound. Ketones are depicted in orange and green, aldehydes in pink and purples, and pyrazines in turquoise.

Commercial natto has a distinct aroma profile [54,55]. Therefore, it is an important parameter in determining natto quality. Volatile organic compounds (VOCs) were determined using GC-MS, and in total 28 compounds were detected and identified (Fig. 7). Boiled unfermented legumes were used as control and contained low amounts of VOCs. None of the strains fermenting brown beans produced a significant increase in total VOC. However, green peas and chickpeas fermented by all strains produced significantly more VOCs compared to their unfermented counterparts. The highest increase in VOCs was observed in green peas fermented with strain 2.8. The effect of strain on VOC production was dependent on the legume. In lupin natto only fermentation with strain 92 and 1.8 increased the total amount of VOCs, while for red lentils a significant increase in VOC was obtained with strain 1.8 and 2.8, but not with strain 92. Generally, the factor “legume” had a larger effect on the VOC profile compared to the factor “strain”. VOC profile of fermented legumes with low total VOC area were characterized by high proportions of acetone (pungent odour), 2-penthyl-furan (beany odour), and aldehydes such as benzaldehydes (almond-like odour) and butanal (pungent odour). Ratios of the different compounds within the VOC profile were similar in fermented lupins, chickpeas, red lentils, and green peas (characterized by high total VOC area). Their VOC profile was largely composed of pyrazines, 2,5-dimethyl-pyrazine and to a lower extent trimethyl-pyrazine and 3-ethyl-2,5-dimethyl-pyrazine. VOC profile of fermented soybeans and brown beans, characterized by lower total VOC area, were made up of ketones, mainly acetoin.

## 4 Discussion

Overall, all the selected substrates, besides for brown beans, were considered appropriate for the development of a novel natto. All fermented brown beans developed a worst texture and were low in vitamin content as well as in FAA and VOCs. It is possible that the presence of the hull in brown beans caused a lower accessibility of the nutrients to the bacteria, which were not able to develop as many secondary metabolites, resulting in a suboptimal product.

We observed that the factor “strain” as well as the factor “substrate” affected the development of the PGA strands, despite the observation that the difference in microbial growth between samples was not significant. Substrates fermented with strain 2.8 seemed to develop more PGA, especially when compared to products made with strain 92. The latter fermented product that did not show the development of abundant strands, besides for the chickpea sample, which seems to be the substrate that on average showed more PGA. These conclusions are however based on visual observation. To have a more quantitative assessment of PGA formation, the viscosity of natto by rheological analysis could be considered.

Natto was inoculated with approximately 5 log_10_ CFU/g of *B. subtilis* in all the tested substrates and the bacterial cell count after maturation reached values around of at least 7.6 log_10_ CFU/g. The difference in growth after maturation was not significant between the tested strains and substrates. This indicates that all the substrates and strains assessed are adequate to ensure sufficient microbial growth, also when compared to natto made from soybeans as reference. Compared to other studies, the bacterial cell counts found in our natto variants is at the lower end. Wei [45] reported viable cell counts in the final product between 8.6 and 9.8 log_10_ CFU/g. These higher microbial cell counts could be attributed to (I) the 10 to 100-fold higher inoculation that was used, (II) the longer steaming times of the substrates and finally (III) strain differences.

Concerning overall alkalinization strain 2.8 was the only one that did not deliver a significant increase in pH during fermentation on any of the substrates, and at the same time it was also the greatest PGA producer, especially in chickpeas natto, and reached at least 8 log_10_ CFU/g in all the substrates, besides for lupin. Pradhananga [56] reported that their natto made from soybeans had a final pH of about 7.6 after fermentation, while Wei [45] reported that natto their had a pH between 7 and 8.1 after 20 h of fermentation, depending on the cooking time and strain used. Our findings are in line with these reported in literature; indeed, all the samples had a pH ranging from 6.7 to 8.1.

Phylloquinone (vitamin K_1_) is a vitamin of plant origin, indeed it was found in all our fermented and unfermented samples. Fermentation did not affect the content of this vitamin which stayed unchanged. Soybeans had the overall highest vitamin K_1_ concentration of approximately 20 μg/100 g of product. It has previously been reported that dried soybeans contain approximately 37 μg/100 g of phylloquinone [57]. In the other legumes we measured a lower content of vitamin K_1_ compared to soy. Dried chickpeas and dried lentils have been reported to contain 31 μg/100 g, and 34 μg/100 g of phylloquinone [58], which is higher than our values. This difference in vitamin K_1_ compared to literature is likely due to water absorption during soaking and cooking.

Menaquinone is an essential co-factor in many metabolic reactions of *B. subtilis*. This microorganism has all the genes that encode for the enzymes of the menaquinone biosynthesis pathway. This vitamin is constituted by a naphthoquinone unit with isoprenyl sidechains of various lengths. The length of the isoprenyl side-chain is indicated by *n* in MK-*n* [59]. Natto is notoriously rich in vitamin K2, indeed it is one of the richest sources of this vitamin among food products. While animals produce exclusively MK-4, each bacterial species produces different menaquinones with different length of isoprenyl sidechains. *B. subtilis* produces exclusively MK-7. Therefore, in this study MK-4 was used as an internal standard to establish the extraction yield of the microbially produced MK-7. Commercial natto contains approximately 775 μg/100 g [23] to 865 μg/100 g of MK-7 [60], while the highest content of MK-7 in soybeans natto, produced in this study with strain 92, was at best approximately half the commercial reported values. However, natto made with red lentils had either comparable amount of MK-7, or double compared to the commercial references here reported. The highest amount of MK-7 was found in chickpeas natto produced with strain 92, in which vitamin K_2_ was almost three times higher than the reported values for standard natto. In this specific sample, the vitamin was even higher than the amount found in a natto obtained with a *B. subtilis* mutant overproducing vitamin K_2_. This mutant was obtained by analogue resistance to intermediates of the shikimate pathway, and UV exposure [61], in which the amount of MK-7 was 1719 μg/100 g. These results indicate that the production of MK-7 is both strain and substrate dependent, and that these factors have significant interaction. Consequently, by selecting the appropriate strain and substrate, it is possible to have a natto with equal or even higher content of vitamin K_2_ compared to the one currently available in the market. The relevance of this finding is enhanced by the well-establish health benefits of vitamin K_1_ and K_2_, which are proven to contribute to bone and vascular health in humans [62].

Fibrinolytic activity was quantified by the clear zone on fibrin plates (5.0–23.0 mm) as an indicator for the presence of nattokinase (10–1000 FU/mL). Chickpea, red lentils and soybean fermented with strain 1.8 and 2.8 showed fibrinolytic activity producing a clear zone between 3.7 and 11.0 mm. Therefore, fibrinolytic activity of these fermented samples is likely to fall in the range of 7–350 FU/g. These results are in a similar range compared to earlier work on soybean natto, which observed 130 FU/g [63]. Significantly higher nattokinase concentrations were observed in soybeans fermented with strains 1.8 and 2.8 compared to the average. Soybeans fermented with strain 2.8 contained the highest concentration (347 FU/g). Studies on metabolic pathways of *B. subtilis* have found a positive correlation between the addition to the growth medium of soy peptone, watery, or ethanolic extracts of soy as nitrogen sources and nattokinase production [64,65]. In our study, a high nattokinase concentration of 323 FU/g, was observed in nutrient broth inoculated with strain 2.8, which contains meat extract, yeast extract, and peptone. Different studies observed that these are excellent nitrogen sources for high nattokinase production by *B. subtilis* [[64], [65], [66]]. Additionally, Man and co-workers [65] observed that glycine addition showed a positive effect on nattokinase production, while addition of glutamate, asparagine, arginine, and serine decreased nattokinase production. In our results, fermented soybeans showed lower concentrations of all these amino acids, compared to lupins, chickpeas, and red lentils. The combination of the results by Man and co-workers [65] and our finding is a strong indication for the presence of a negative feedback mechanism between increasing concentrations of these amino acids, and the productivity of nattokinase. Therefore, the lower FAA content in fermented soy could play a role in the high nattokinase concentrations observed.

Fungi, plants, and bacteria produce thiamine. Its deficiency in humans can lead to severe metabolic disfunctions, and death [16]. Thiamine is also well known for its role in development of meat flavour during cooking, indeed upon heat-degradation the thiazole subunit forms many sulphur containing compound that strongly contribute to the aroma of cooked meat [15]. In *B. subtilis*, thiamine plays a role in many biological processes, such as the central carbon metabolism, the metabolism of amino acids, and even vitamin K_2_ production [67]. Thiamine biosynthesis in *B. subtilis* is strictly regulated by riboswitches, present in the three single genes and two operons encoding for enzymes involved in thiamine biosynthesis, transport, and salvage. The riboswitches located upstream of the open reading frames (ORFs) bind with the target ligand, thiamine pyrophosphate, and forms intrinsic transcription terminators, and therefore repress the production of thiamine [68]. Since the *de novo* biosynthesis of thiamine is an energy expensive process for *B. subtilis*, its uptake is preferred when the molecule is present in the fermentation substrate. In this case the unchanged thiamine concentration before and after fermentation is expected since *B. subtilis* is not expected to produce thiamine when it is present already. Accordingly, the only effect that the microbial activity had on thiamine, was the observed conversion of thiamine to the biologically active form thiamine pyrophosphate, which increased in proportion in all the fermented legumes, exception for soy fermented with strain 2.8 and 92. The amount of thiamine found in cooked and fermented substrate is in the same order of magnitude compared to the reported literature values (5–9 mg/kg) [69,70]. But still too low in order to deliver a meat flavour upon heat exposure [71].

*B. subtilis* is well known for its capacity of secreting extracellular proteases [72,3]. These enzymes are responsible for the breakdown of proteins and release of free amino acids which drives the subsequent release of ammonia in natto. Free amino acids are an important parameter in understanding extracellular protein degradation and subsequent production of secondary metabolites [73]. Fermentation driven by *B. subtilis* leads to release of free amino acids followed by an increase in the extracellular pool size of amino acids in lupins, chickpeas, red lentils. This means that the uptake of released amino acids by *B. subtilis* is slower than the rate of release of amino acids driven by extracellular protein hydrolysis. Similar free amino acid profiles were observed for all strains in fermented lupin, chickpea, red lentil, and green pea (Fig. 6), indicating that similar proteolytic enzymes are excreted, and the same amino acids are utilized, independently of the strain used. These similar profiles suggest that identical protein catabolism pathways were utilized in all strains. Net consumption of FAA during fermentation was only observed in glutamine/arginine. A result that can be explained by the fact that these amino acids are the preferential nitrogen sources for *B. subtilis* [74]. Secondly, γ-PGA production has been observed to result in consumption of glutamine, among others [4,75]. A larger increase in total FAA was observed in fermented lupins, chickpeas, red lentils, and green peas, was higher compared to the control soybeans. This was a sign of high proteolytic activity, which is an indication of a superior ability of *B. subtilis* to metabolize proteins in these substrates.

Ammonia is an integral part of natto aroma perception [5]. Its acceptance by consumers differs based on cultural background and familiarity with the product [76]. High ammonia concentrations were found in samples with a high pH. A correlation which can be explained by the high pKa of ammonia, which is 9.24 [77]. These results are in accordance with findings from Kada an co-workers [5], who observed a positive correlation between contents of free L-glutamate, L-alanine, L-glutamine, L-arginine and L-aspartate and ammonia production in *B. subtilis* var. natto fermentation of soybeans. In this study they observed that ammonia content of natto decreased by half when glutamate dehydrogenase was inactivated, indicating that this step is one of the major ammonia-releasing reactions in natto fermentation. Furthermore, ammonia is a precursor in the formation of pyrazines [78,79]. High ammonia concentrations were observed in samples with high total FAA, these compounds were released in similar pathways, which explains their co-production [80]. This positive correlation was observed in our results, as well, with higher concentrations of ammonia found in red lentils, lupins, and green peas (Fig. 5B). These legumes similarly contained highest concentrations of ammonia of all substrates (Fig. 5B).

**Fig. 8:**
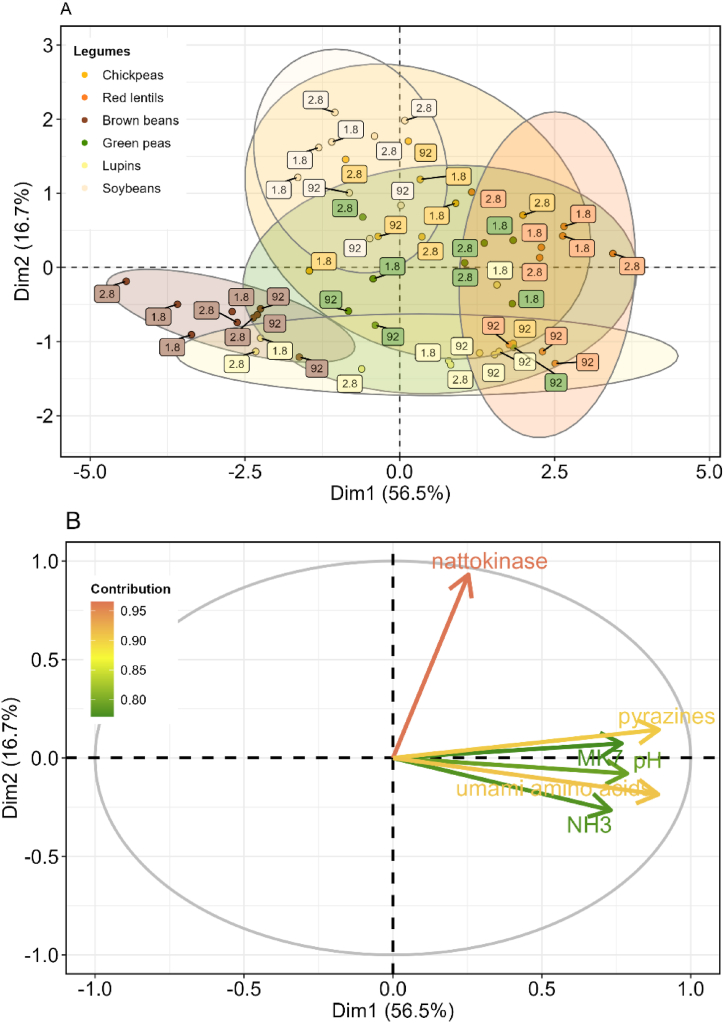
Principal component analysis (PCA) plot showing the separation of legume samples based on their metabolic profiles. (A) Individuals plot of the first two principal components, Dim1 (56.5%), and Dim2 (16.7%), with each point representing a sample coloured by legume. Ellipses indicate a 95% confidence interval around each group. (B) Loadings plot showing the contribution of each metabolite to the principal components, with each loading vector coloured by its magnitude. The metabolites with the largest contributions are labelled.

Natto is a very aromatic product [81]. Major contributors to natto aroma are ketones and pyrazines [12], [11]. The main ketone observed was acetoin (3-hydroxybutanone). Acetoin has a high odour threshold but is readily oxidized to diacetyl (2,3-butanedione), with an odour reminiscent of butter or cream [82]. These compounds were present in the highest concentration in fermented soybeans and brown beans (Fig. 7). Secondly, acetoin can be non-enzymatically transformed to 2,3,5,6-tetramethylpyrazine [83]. Pyrazines are heterocyclic aromatic compounds, with a high flavour dilution value, and an odour reminiscent of roasted nuts [11]. Chen [54] observed that these non-enzymatic reactions in the formation of pyrazines occur later in the fermentation process, therefore, a high contribution of pyrazine indicate a mature fermented product. Pyrazines were the major contributor to the VOC profile of fermented lupins, chickpeas, red lentils, and green peas. Similarly, to fermented legumes, pyrazines were also a major factor in aroma profile of roasted soybeans [84]. 2,5-dimethylpyrazine was present in the highest amount. L-threonine serves as a substrate for 2,5-dimethylpyrazine formation [6,7]. This explains why free L-threonine concentrations remain low in fermentations with high pyrazine formation (Figs. 6, 7B and 8).

Principle component analysis (PCA) was used to examine parameters affected by fermentation of our data (Fig. 8). Samples were grouped by legume type and plotted in Dim1 and Dim2 (Fig. 8A). Dim1 represented 56.5% of the variability, while Dim2 represented 16.7%. Loading vectors were coloured by magnitude of contribution (Fig. 8B). All variables were well represented in these two dimensions. Samples clustered well by legume type, but much overlap was observed, especially among soybeans, lupins, green peas, and red lentils (Fig. 8A). Clustering by strain was less clearly observed, indicating that the effect of legume choice is larger than that of strain. However, this is also indicative of the large biological variation among samples. All parameters were highly positively correlated, except for nattokinase. Since high values for all parameters included in this PCA are desired natto characteristics, samples positively correlating with these parameters are the most promising for natto production. Red lentils fermented with strain 1.8 and strain 2.8 most consistently correlate with all parameters (Fig. 8A and B). Therefore, we can conclude that red lentils can be a promising alternative to soybeans, for natto production, regarding both product quality and nutritional benefits.

## 5 Conclusions

Brown beans were the only substrate that led to a final product which had lower vitamin content, aroma compounds, and PGA, compared to soybeans natto. In particular, the low free amino acid content and VOCs indicated a slower, thereby incomplete fermentation. While traditional soybean natto contained highest vitamin K_1_, and fibrinolytic activity, red lentils, green peas, chickpeas, and lupins outperformed soybeans as substrate for natto in terms of alkalinization, free amino acid content (FAA), ammonia content, total volatile organic compounds (VOCs), proportion of pyrazines, and vitamin K_2_ content. Natto made with green peas and inoculated with strain 2.8 had highest levels of VOCs. Fermented red lentils also contained highest vitamin K_2_ and B_1_ content, with menaquinone being two-fold higher compared to commercially available natto. While natto made with chickpeas inoculated with strain 92 had the highest vitamin K_2_ content of 2 mg/100 g of product, three-fold higher compared to commercially available natto. This study shows that the production of natto using alternative legumes to soy is possible, but that the overall quality, regarding the texture, aroma, and vitamin content is determined by the interaction between the strain and legume.

## Funding statement

This research is part of the project B-Twelve Insight, which is co-financed by Top Consortium for Knowledge and Innovation Agri & Food by the Dutch Ministry of Economic Affairs. The project is registered under contract number TKI AF18081.

## CRediT authorship contribution statement

**Rebecca Rocchi:** Writing – original draft, Visualization, Methodology, Investigation, Formal analysis, Data curation, Conceptualization. **Jasper Zwinkels:** Writing – original draft, Visualization, Methodology, Investigation, Formal analysis, Data curation, Conceptualization. **Merit Kooijman:** Validation, Investigation. **Alberto Garre Alberto:** Formal analysis. **Eddy J. Smid:** Writing – review & editing, Supervision, Project administration, Methodology, Funding acquisition, Conceptualization.

## Declaration of competing interest

The authors declare that they have no known competing financial interests or personal relationships that could have influenced the work reported in this manuscript.
