# Supplementary material for: Development of novel natto using legumes produced in Europe
Source: Heliyon. 2024 Feb 29;10(5):e26849. doi: 10.1016/j.heliyon.2024.e26849 (PMC10923668; doi:10.1016/j.heliyon.2024.e26849)
Supplement: Multimedia component 1 [file mmc1.docx]

## Supplementary material


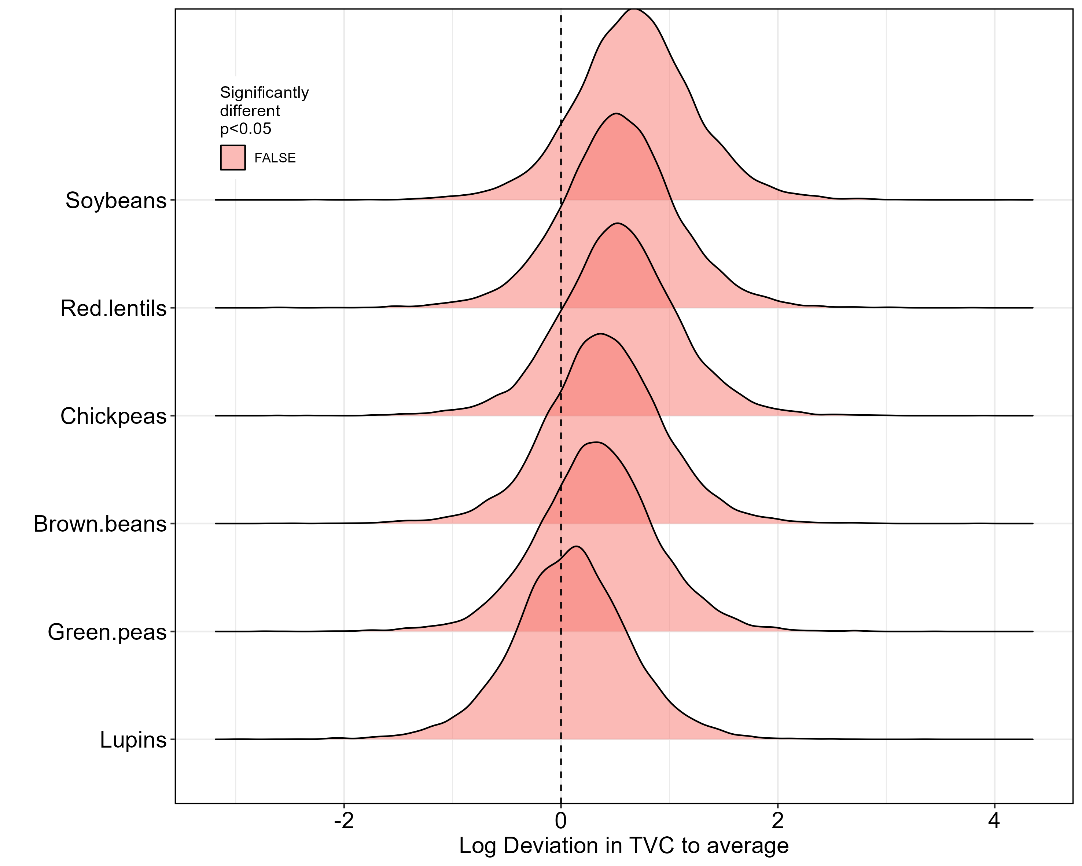
Supplementary material 1-. A posteriori estimates of the difference between the concentration of TVC per each substrate with respect to the pooled average. The colour of the density plots indicates whether differences are significant based on contrast analysis (alpha = 0.05).The differences have been calculated directly from the draws of the Markov Chain.

*Supplementary material 2-* Top *A posteriori* estimates of the difference between the concentration of pH per each substrate with respect to the pooled average. The colour of the density plots indicates whether differences are significant based on contrast analysis (alpha = 0.05). The differences have been calculated directly from the draws of the Markov Chain. Bottom: Contrast analysis of the combined effect strain-product on the pH value with respect to the concentration obtained for control (uninoculated) samples. The colour of the density plots indicates whether differences are significant (alpha = 0.05). The differences have been calculated directly from the draws of the Markov Chain.

Supplementary material 3


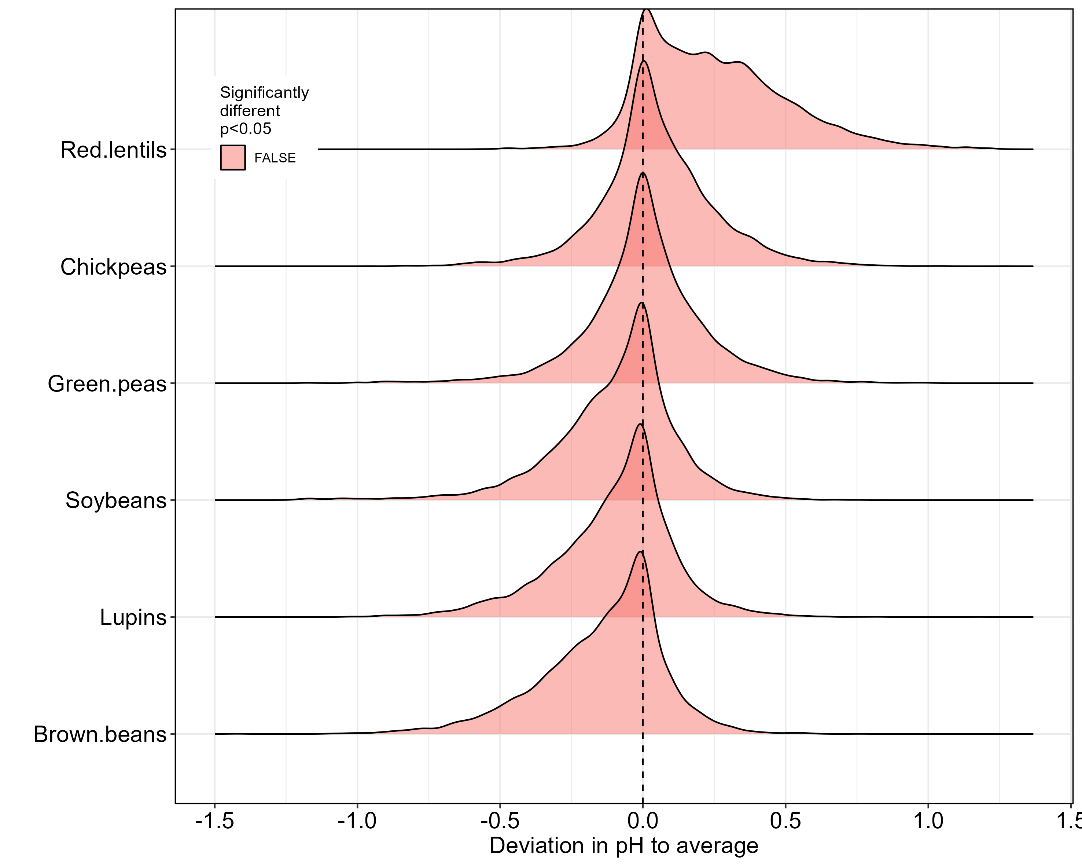

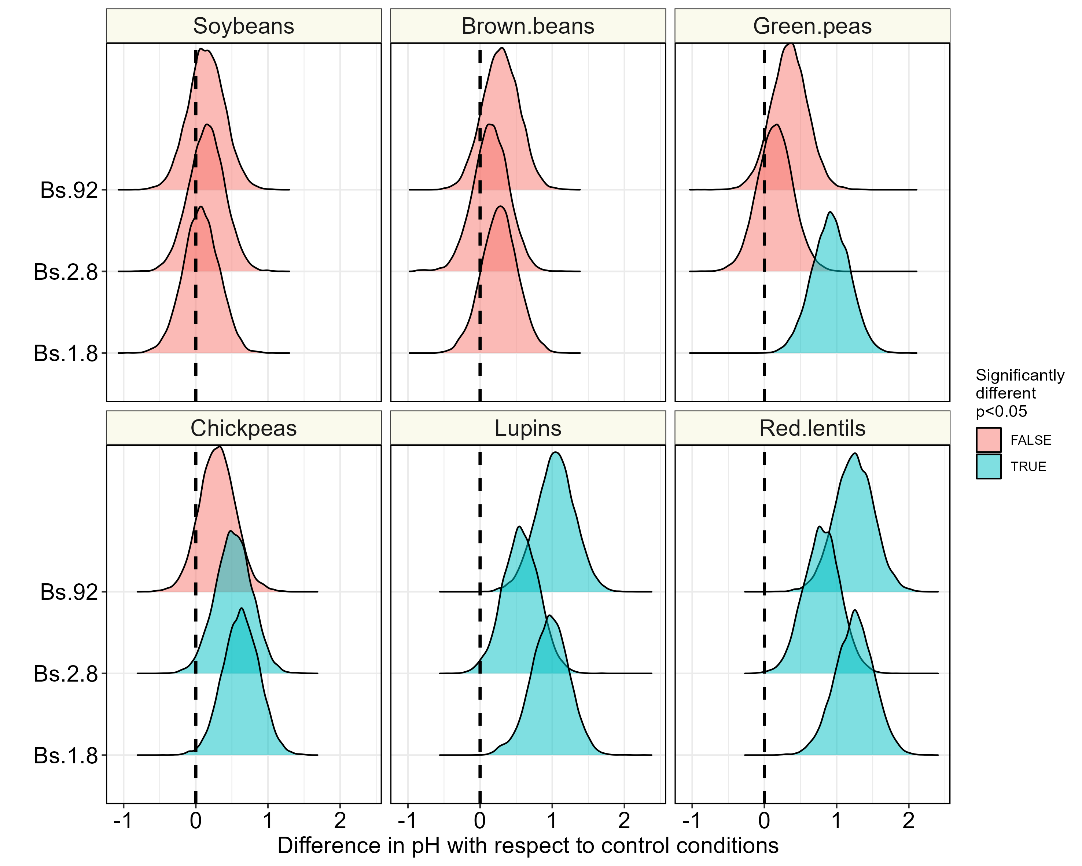


Supplementary material 4- Top A posteriori estimates of the difference between the concentration of thiamine per each substrate with respect to the pooled average. The colour of the density plots indicates whether differences are significant based on contrast analysis (alpha = 0.05). The differences have been calculated directly from the draws of the Markov Chain. Bottom: Contrast analysis of the combined effect strain-product on the concentration of thiamine with respect to the concentration obtained for control (uninoculated) samples. The colour of the density plots indicates whether differences are significant (alpha = 0.05). The differences have been calculated directly from the draws of the Markov Chain.

Supplementary material 5


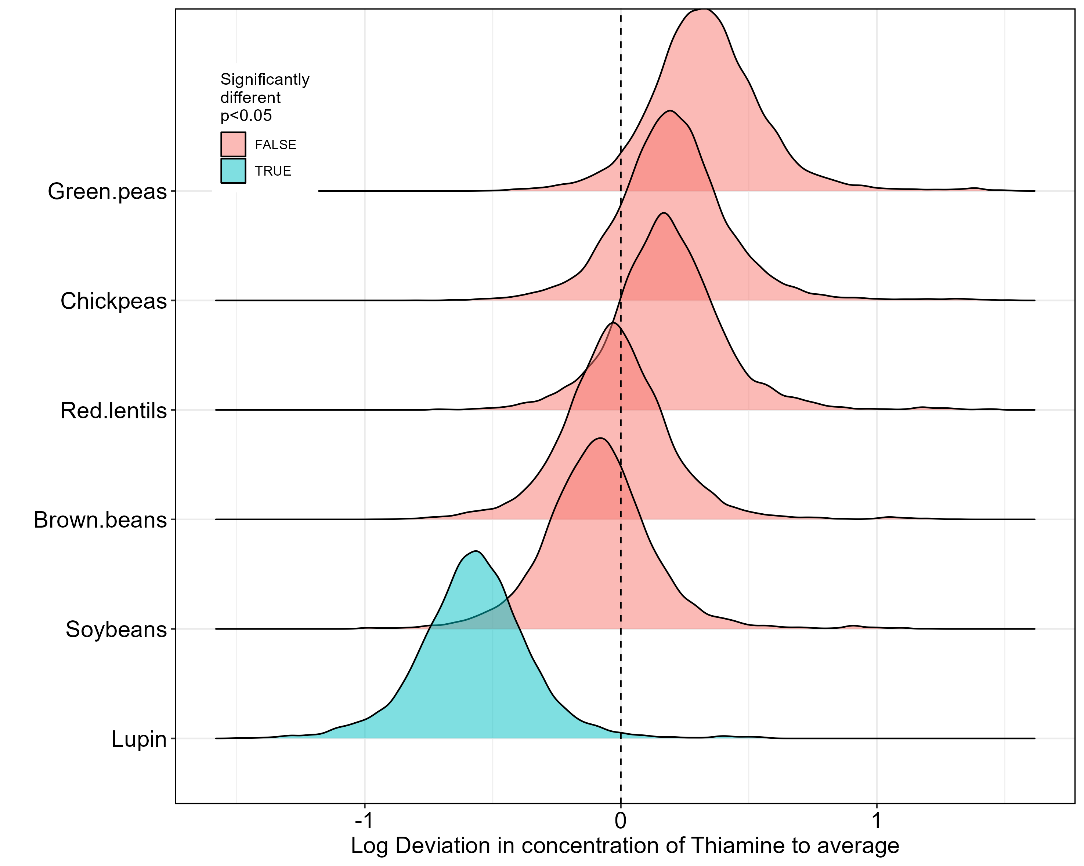

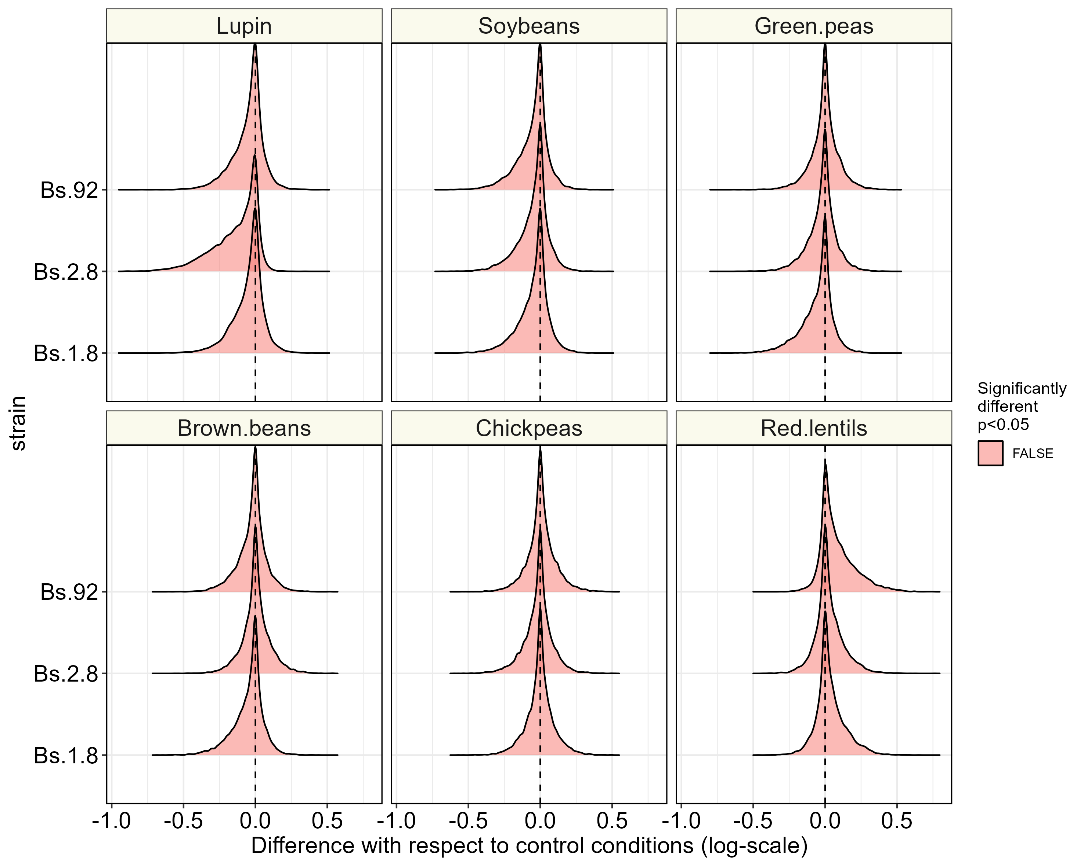


Supplementary material 6 – Top: A posteriori estimates of the difference between the concentration of vitamin K*_1_* per each substrate with respect to the pooled average. The colour of the density plots indicates whether differences are significant based on contrast analysis (alpha = 0.05). The differences have been calculated directly from the draws of the Markov Chain. Bottom: Contrast analysis of the combined effect strain-product on the concentration of vitamin K_1_ with respect to the concentration obtained for control (uninoculated) samples. The colours of the density plots indicate whether differences are significant (alpha = 0.05). The differences have been calculated directly from the draws of the Markov Chain.

Supplementary material 7


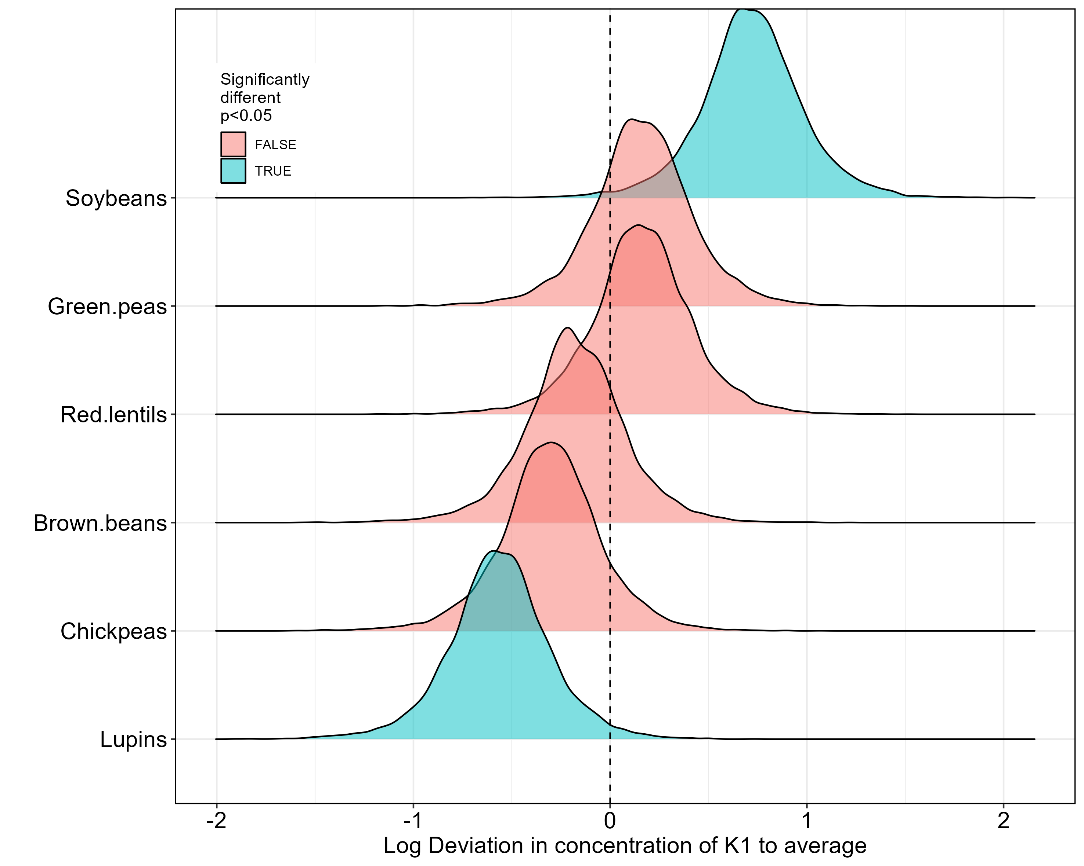

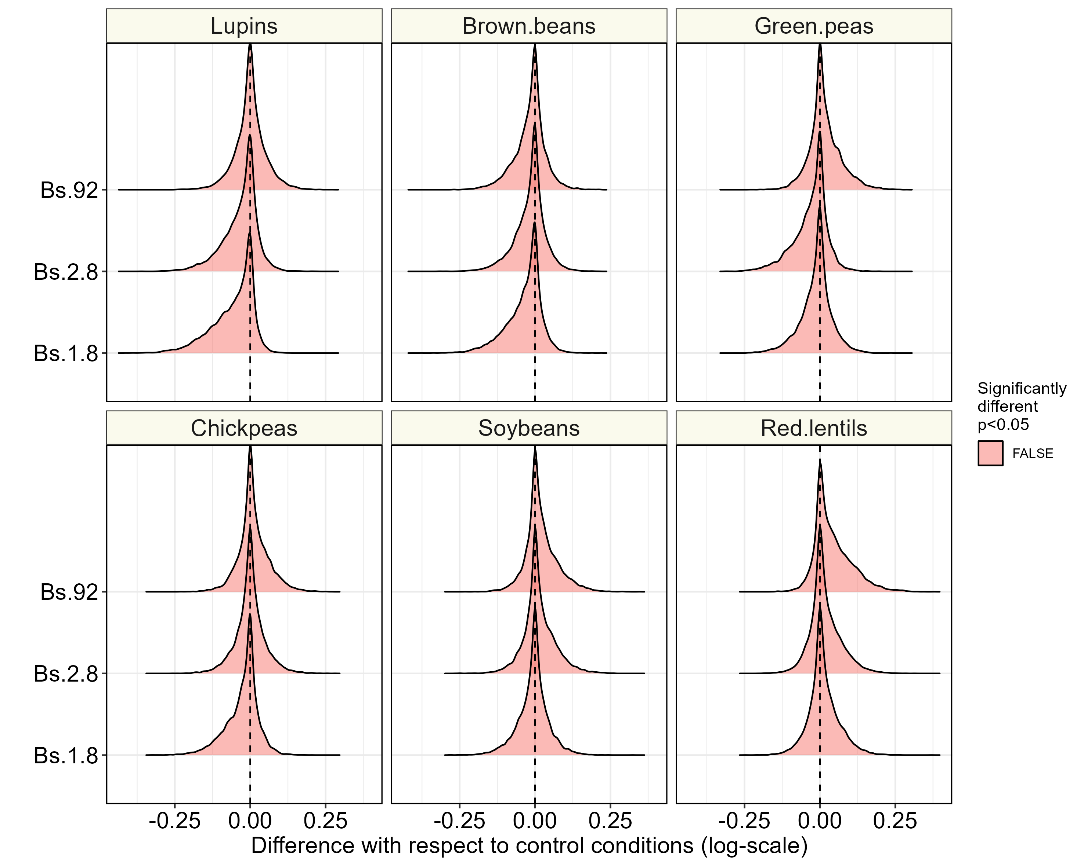


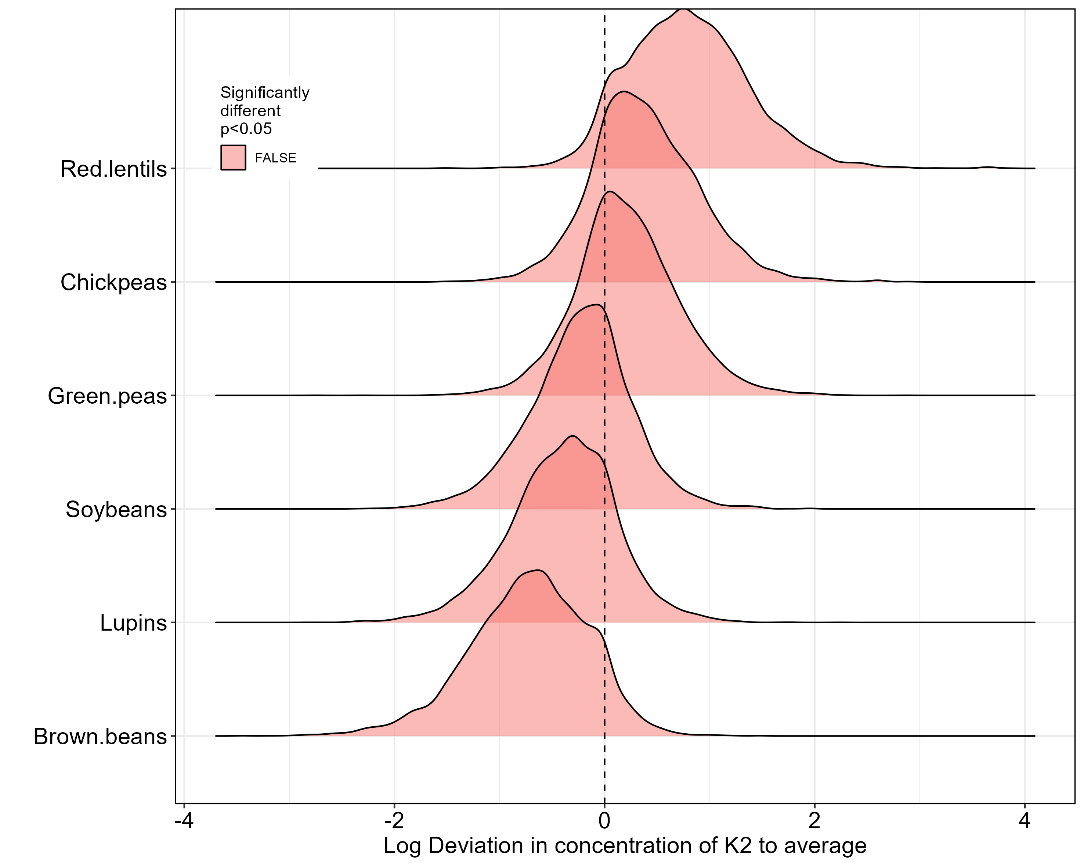


Supplementary material 8- Top: A posteriori estimates of the difference between the concentration of vitamin K_1_ per each substrate with respect to the pooled average. The colours of the density plots indicates whether differences are significant based on contrast analysis (alpha = 0.05). The differences have been calculated directly from the draws of the Markov Chain.


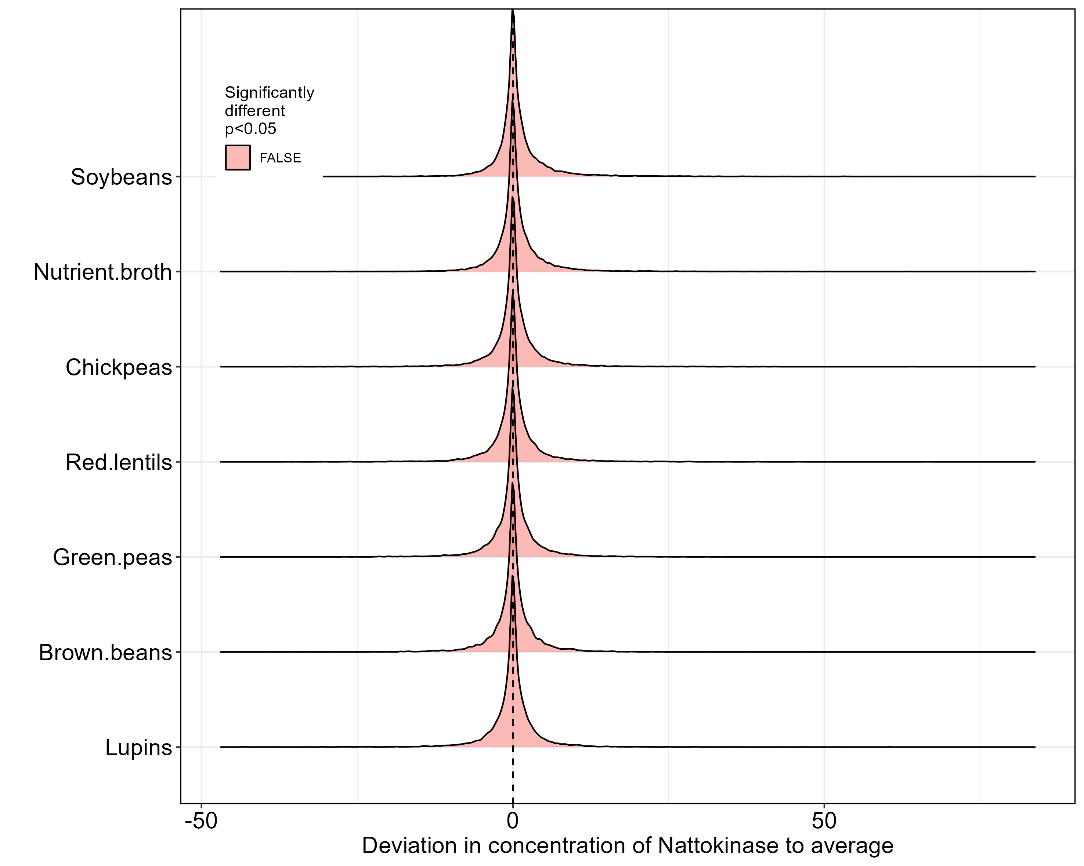

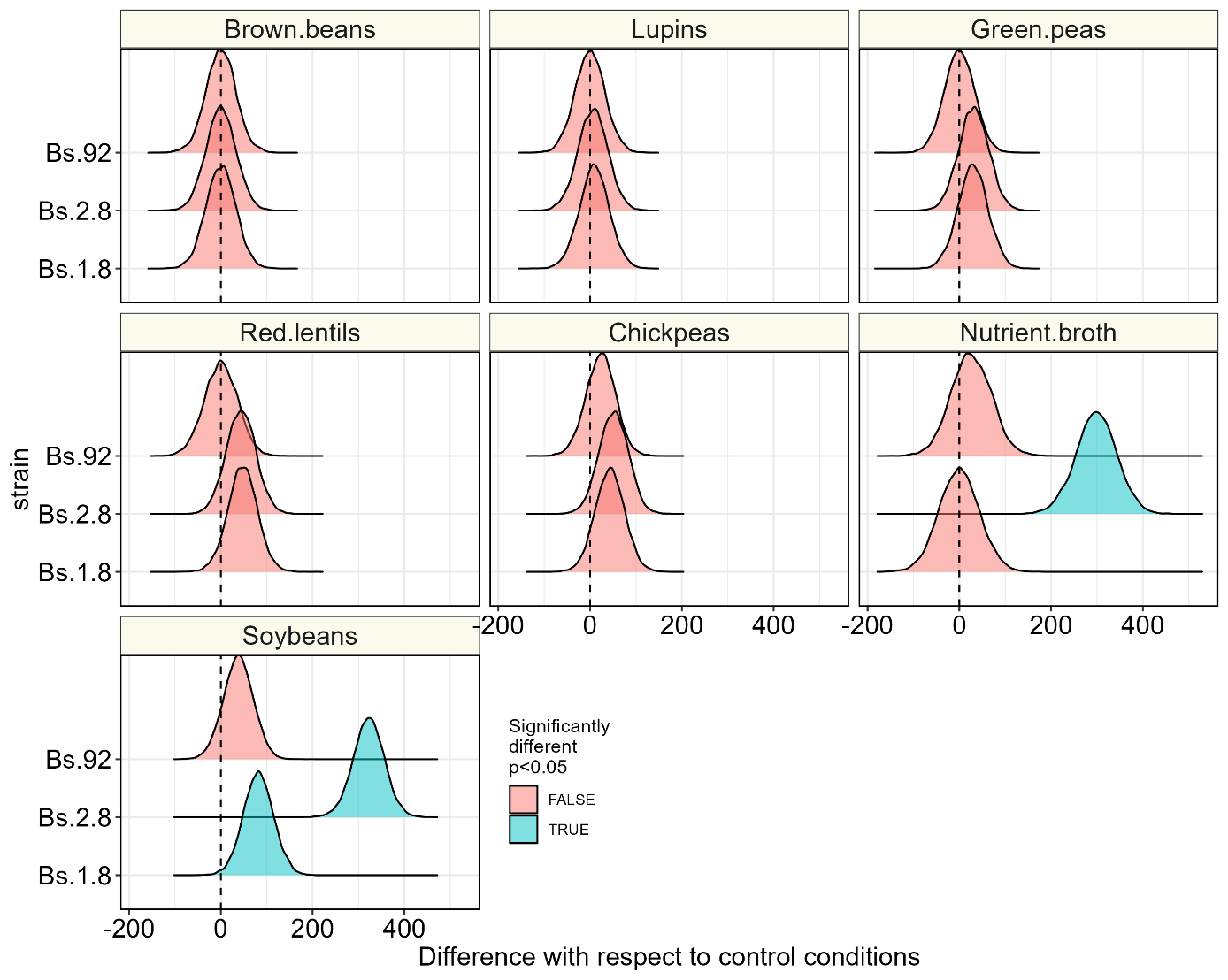


Supplementary material 9 - Top: A posteriori estimates of the difference between the concentration of nattokinase per each substrate with respect to the pooled average. The colours of the density plots indicates whether differences are significant based on contrast analysis (alpha = 0.05). The differences have been calculated directly from the draws of the Markov Chain. Bottom: Contrast analysis of the combined effect strain-product on the concentration of nattokinase with respect to the concentration obtained for control (uninoculated) samples. The colours of the density plots indicate whether differences are significant (alpha = 0.05). The differences have been calculated directly from the draws of the Markov Chain.

Supplementary material 10 - Top: A posteriori estimates of the difference between the concentration of free amino acids per each substrate with respect to the pooled average. The colours of the density plots indicates whether differences are significant based on contrast analysis (alpha = 0.05). The differences have been calculated directly from the draws of the Markov Chain. Bottom: Contrast analysis of the combined effect strain-product on the concentration of free amino acids with respect to the concentration obtained for control (uninoculated) samples. The colours of the density plots indicate whether differences are significant (alpha = 0.05). The differences have been calculated directly from the draws of the Markov Chain.


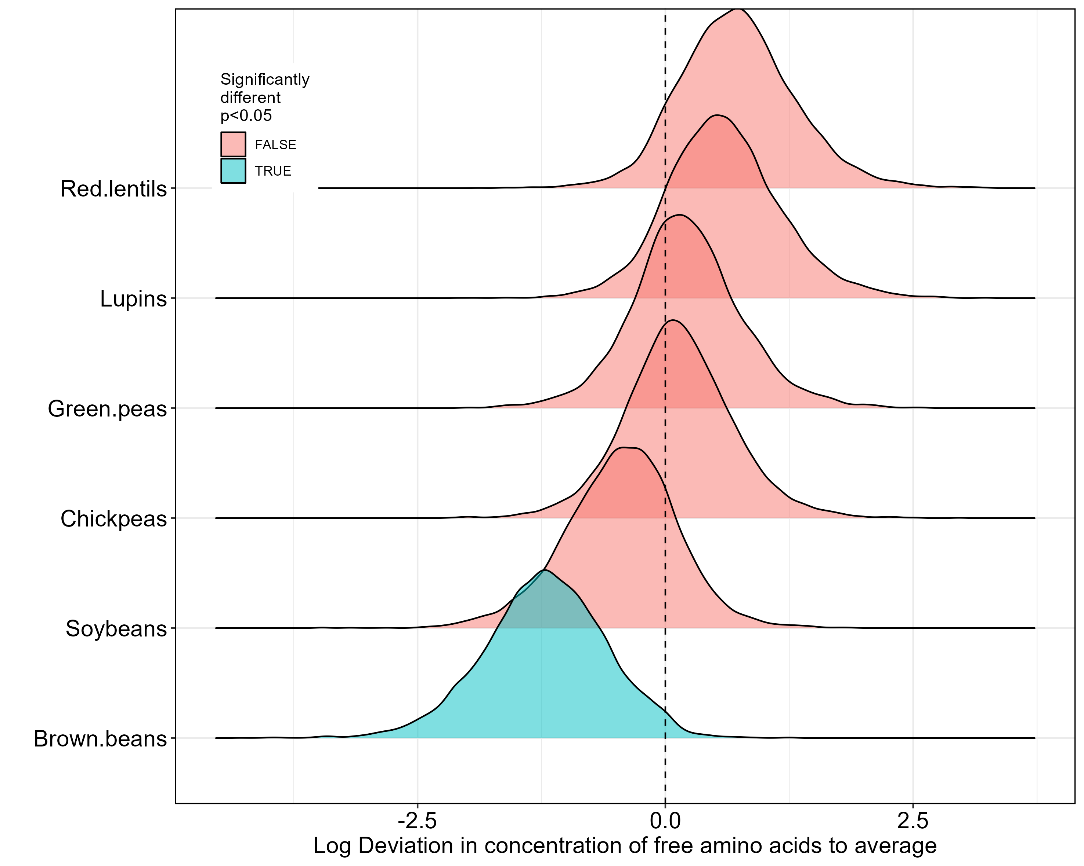

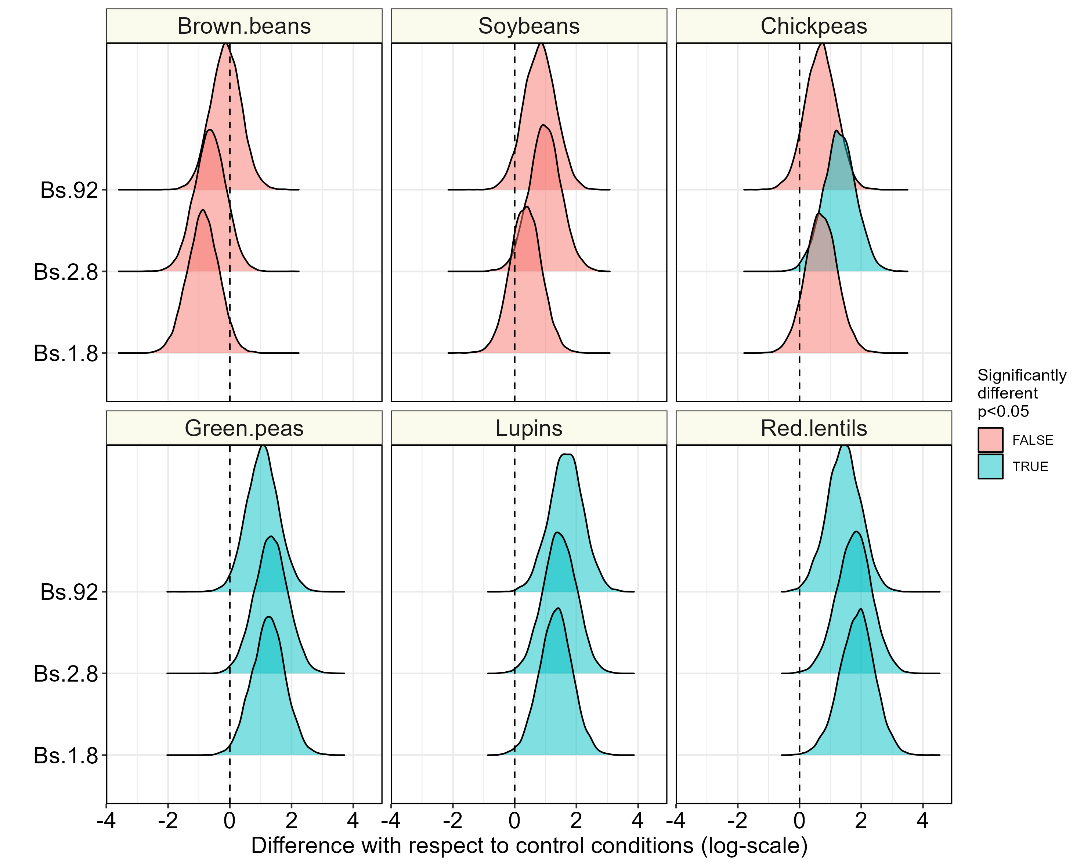


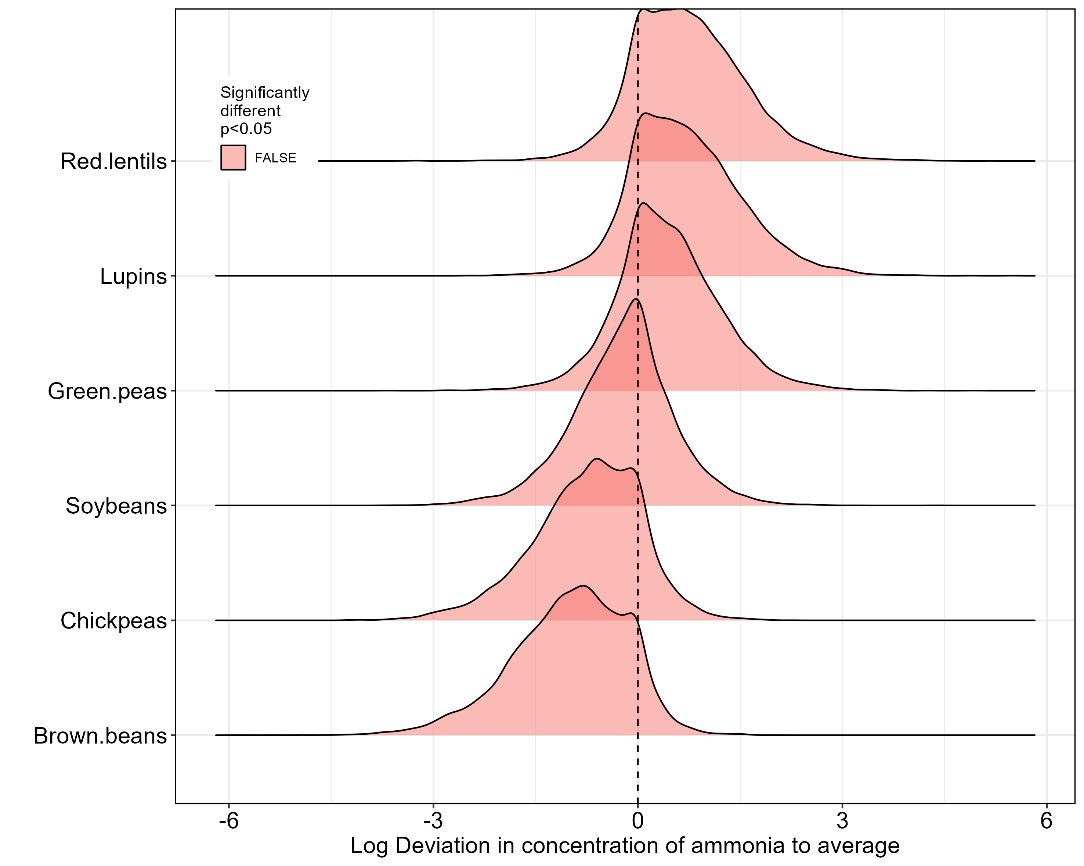

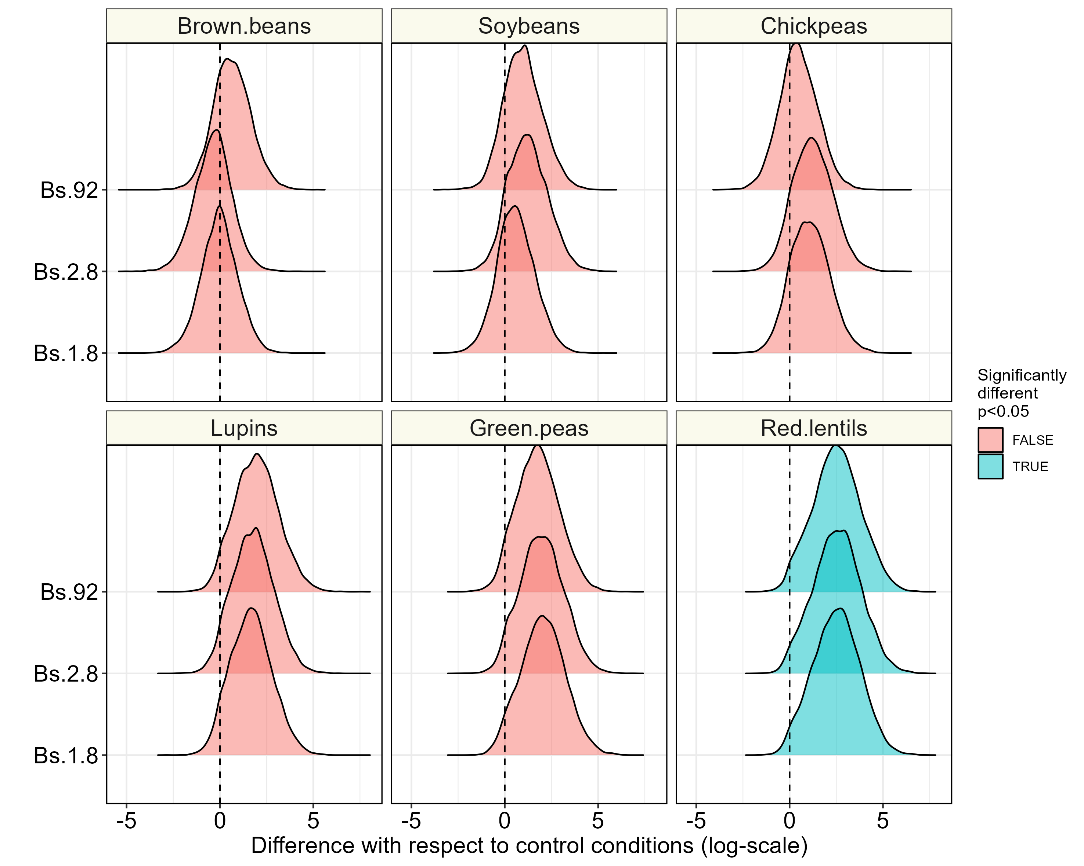


Supplementary material 11- Top: A posteriori estimates of the difference between the concentration of free amino acids per each substrate with respect to the pooled average. The colours of the density plots indicates whether differences are significant based on contrast analysis (alpha = 0.05). The differences have been calculated directly from the draws of the Markov Chain. Bottom: Contrast analysis of the combined effect strain-product on the concentration of free amino acids with respect to the concentration obtained for control (uninoculated) samples. The colours of the density plots indicate whether differences are significant (alpha = 0.05). The differences have been calculated directly from the draws of the Markov Chain.

Supplementary material 12


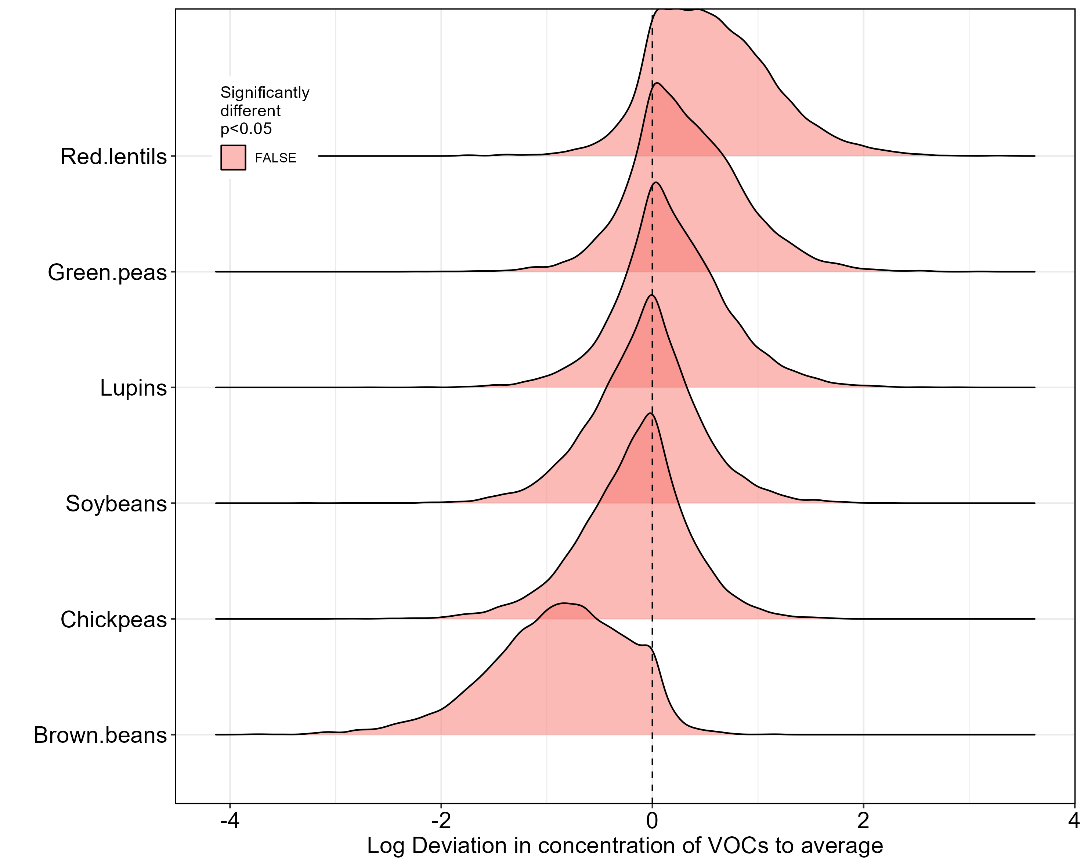

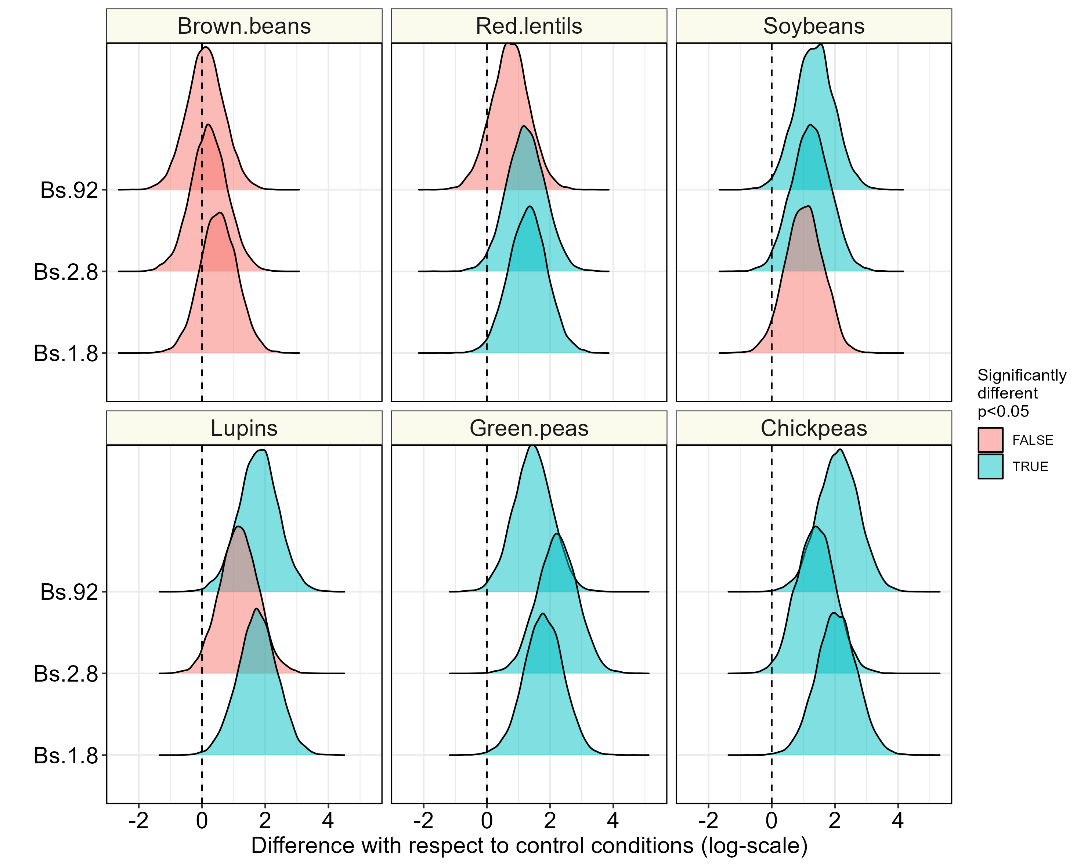


Supplementary material 13- Top: A posteriori estimates of the difference between the concentration of VOC per each substrate with respect to the pooled average. The colours of the density plots indicates whether differences are significant based on contrast analysis (alpha = 0.05).The differences have been calculated directly from the draws of the Markov Chain. Bottom: Contrast analysis of the combined effect strain-product on the concentration of VOC with respect to the concentration obtained for control (uninoculated) samples. The colours of the density plots indicate whether differences are significant (alpha = 0.05) The differences have been calculated directly from the draws of the Markov Chain.

*Supplementary* material 14- Density plot (left) and trace plot (right) of the Monte Carlo iterations for the model parameters estimated from the data on TVC. The plot shows four lines with different colours, indicating four independent runs of the algorithm. The uniform spread of the iterations and the lack of a clear trend in the time series is a support for the convergence of the algorithm.


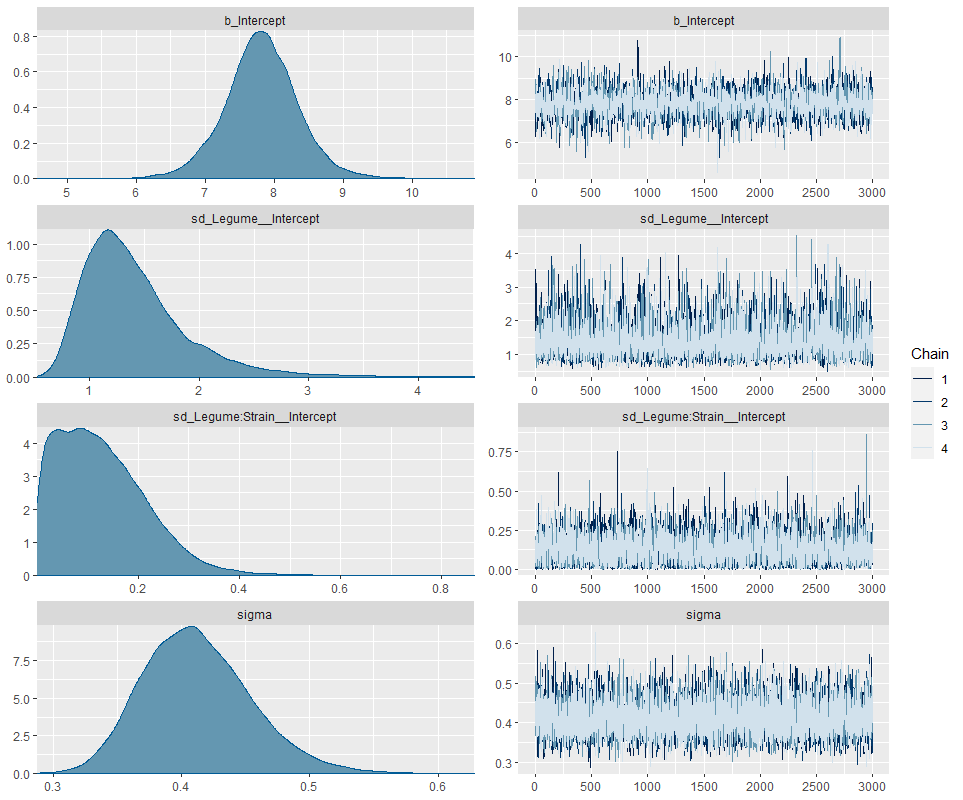


Supplementary material 15- Density plot (left) and trace plot (right) of the Monte Carlo iterations for the model parameters estimated from the data on pH. The plot shows four lines with different colours, indicating four independent runs of the algorithm. The uniform spread of the iterations and the lack of a clear trend in the time series is a support for the convergence of the algorithm.


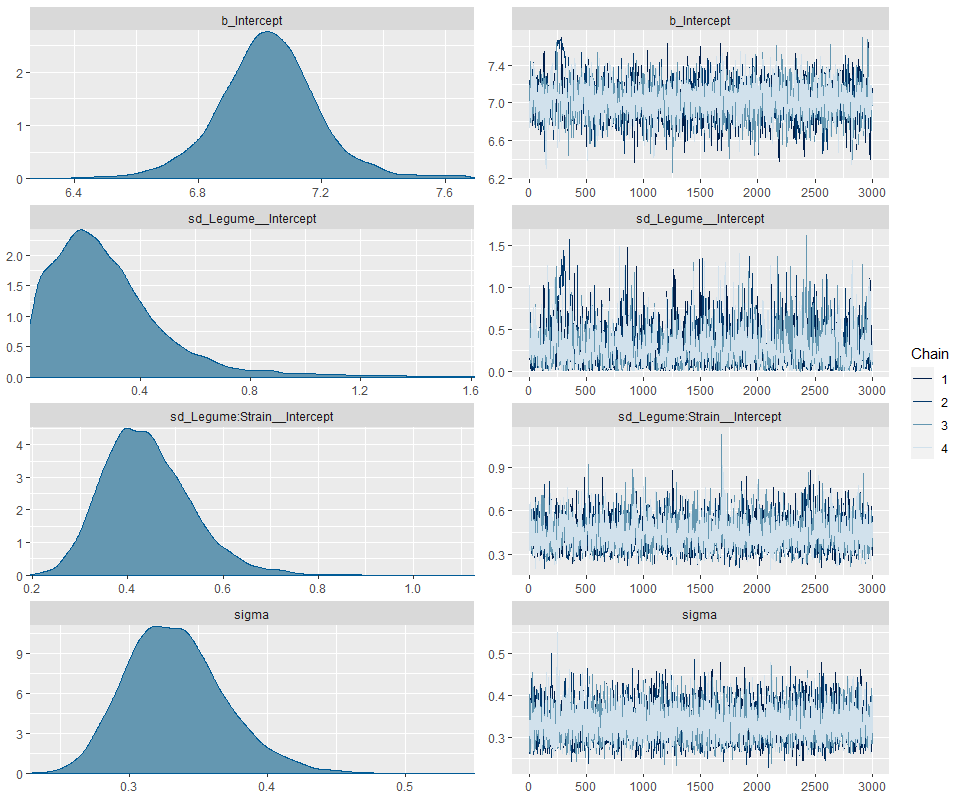


Supplementary material 16- Density plot (left) and trace plot (right) of the Monte Carlo iterations for the model parameters estimated from the data on thiamine. The plot shows four lines with different colours, indicating four independent runs of the algorithm. The uniform spread of the iterations and the lack of a clear trend in the time series is a support for the convergence of the algorithm.


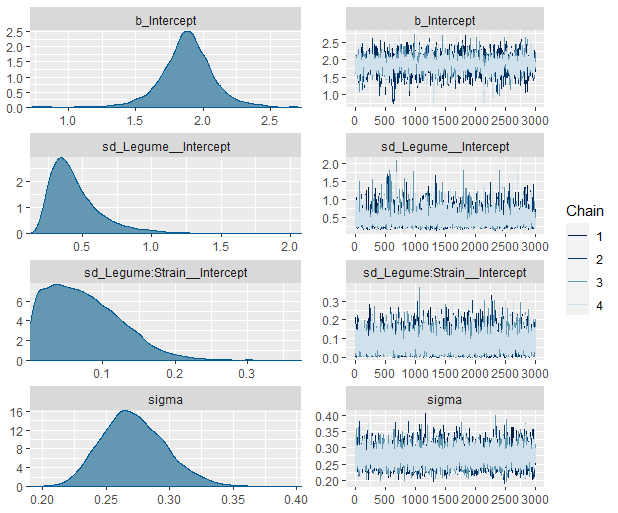


Supplementary material 17- Density plot (left) and trace plot (right) of the Monte Carlo iterations for the model parameters estimated from the data on K1. The plot shows four lines with different colours, indicating four independent runs of the algorithm. The uniform spread of the iterations and the lack of a clear trend in the time series is a support for the convergence of the algorithm.


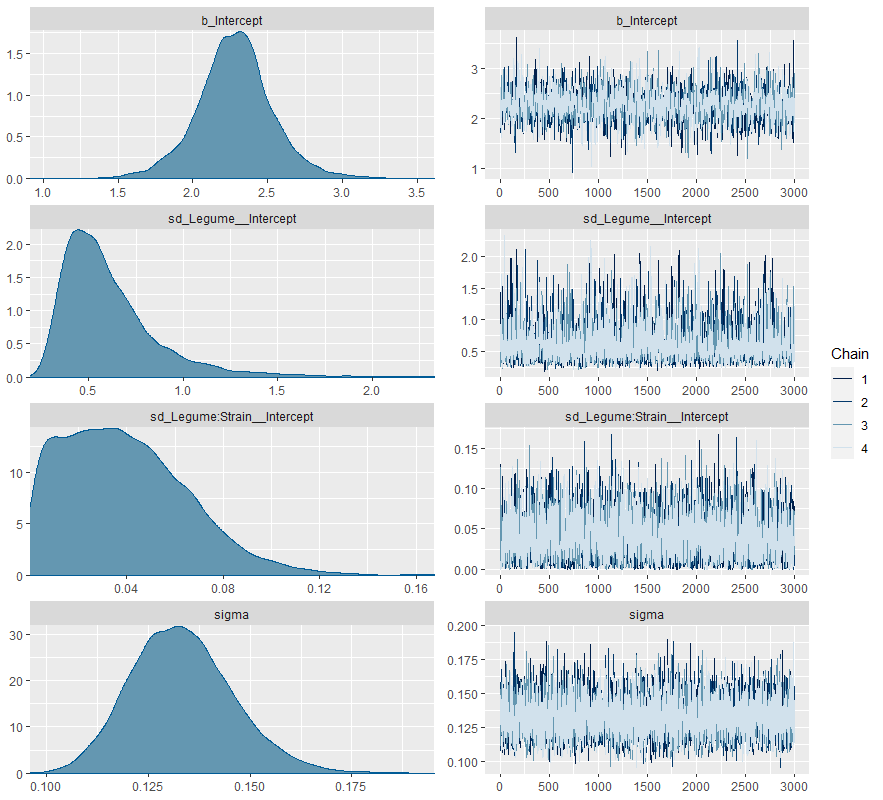


Supplementary material 18- Density plot (left) and trace plot (right) of the Monte Carlo iterations for the model parameters estimated from the data on K2. The plot shows four lines with different colours, indicating four independent runs of the algorithm. The uniform spread of the iterations and the lack of a clear trend in the time series is a support for the convergence of the algorithm.


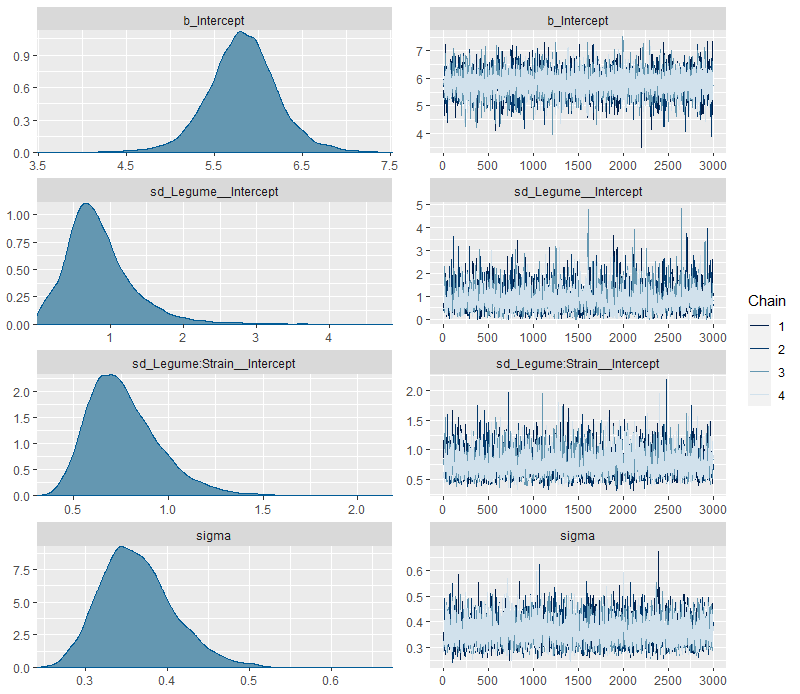


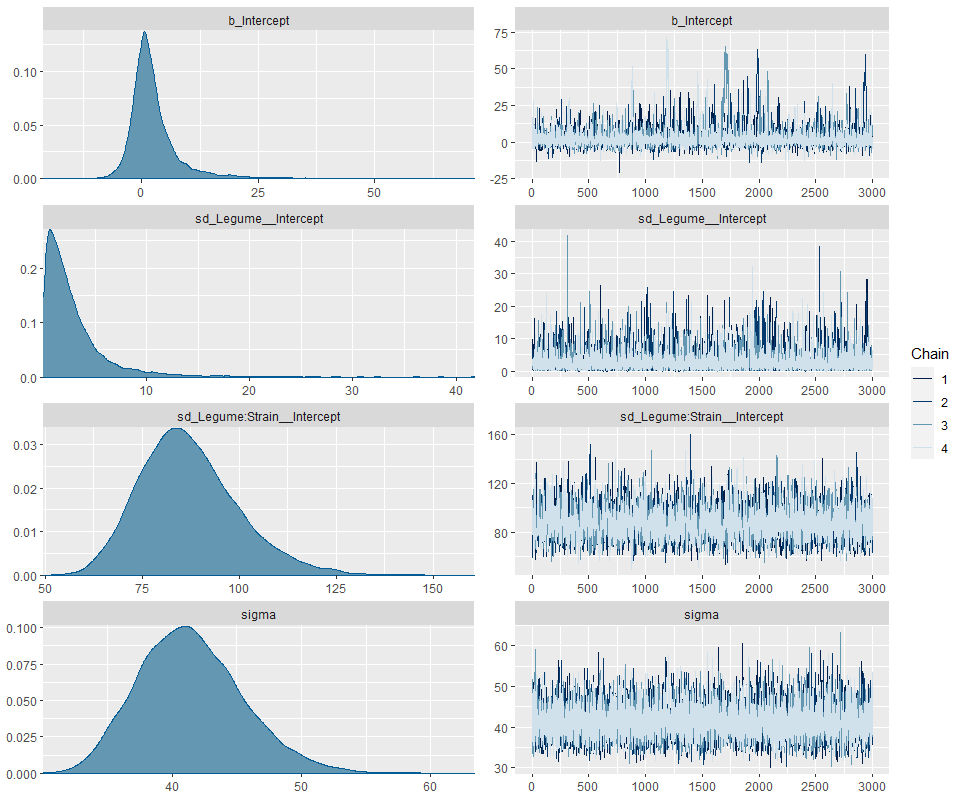


Supplementary material 19- Density plot (left) and trace plot (right) of the Monte Carlo iterations for the model parameters estimated from the data on nattokinase. The plot shows four lines with different colours, indicating four independent runs of the algorithm. The uniform spread of the iterations and the lack of a clear trend in the time series is a support for the convergence of the algorithm.

Supplementary material 20- nattokinase

Supplementary material 21- Density plot (left) and trace plot (right) of the Monte Carlo iterations for the model parameters estimated from the data on the concentration of amino acids. The plot shows four lines with different colours, indicating four independent runs of the algorithm. The uniform spread of the iterations and the lack of a clear trend in the time series is a support for the convergence of the algorithm.


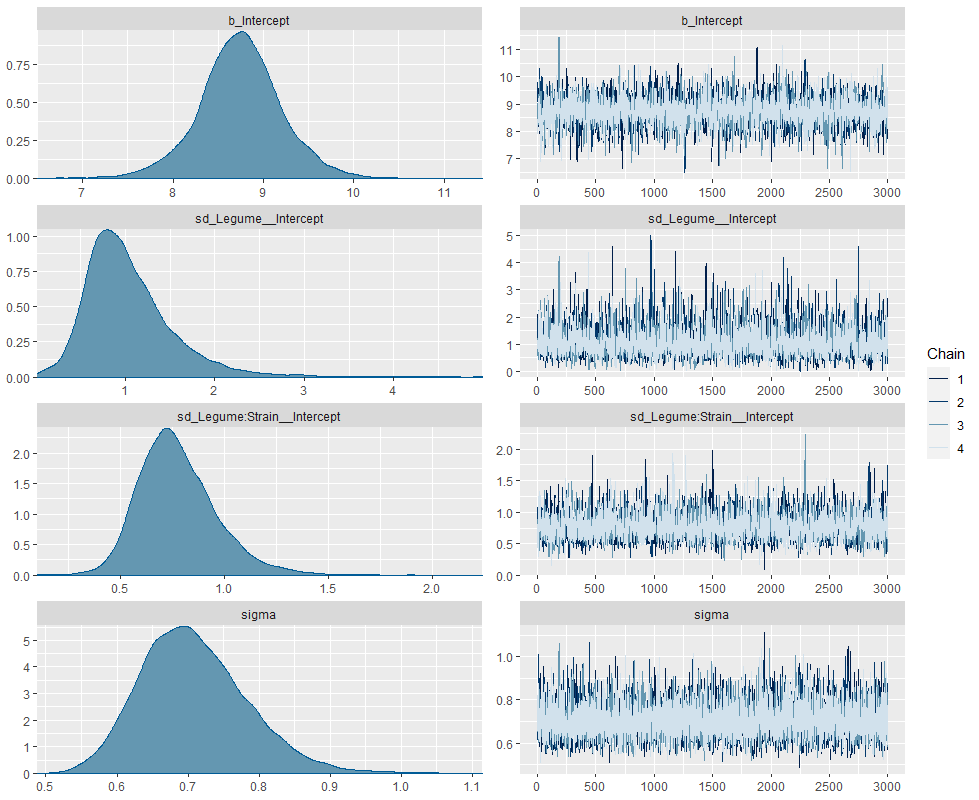


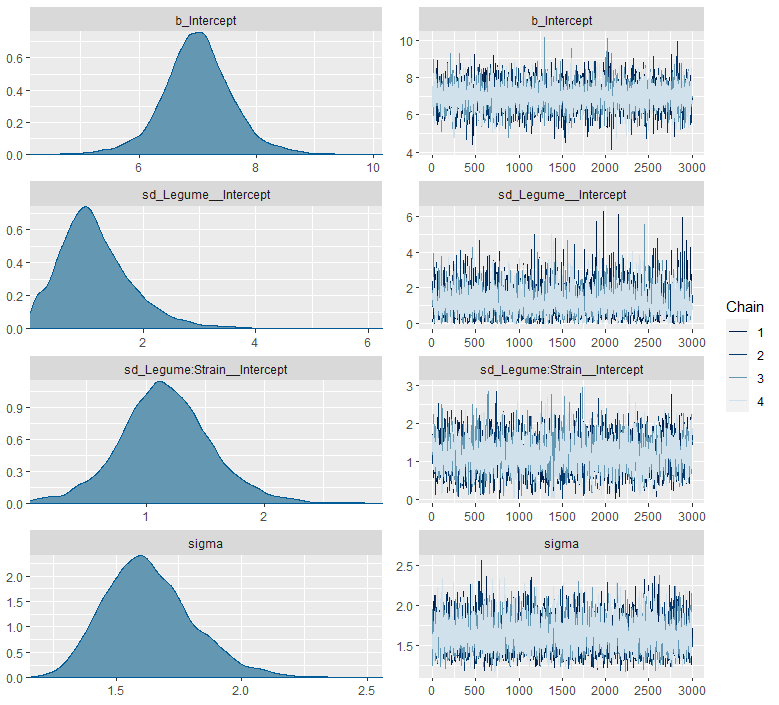


Supplementary material 22- Density plot (left) and trace plot (right) of the Monte Carlo iterations for the model parameters estimated from the data on the concentration of ammonia. The plot shows four lines with different colours, indicating four independent runs of the algorithm. The uniform spread of the iterations and the lack of a clear trend in the time series is a support for the convergence of the algorithm.

Supplementary material 23-ammonia


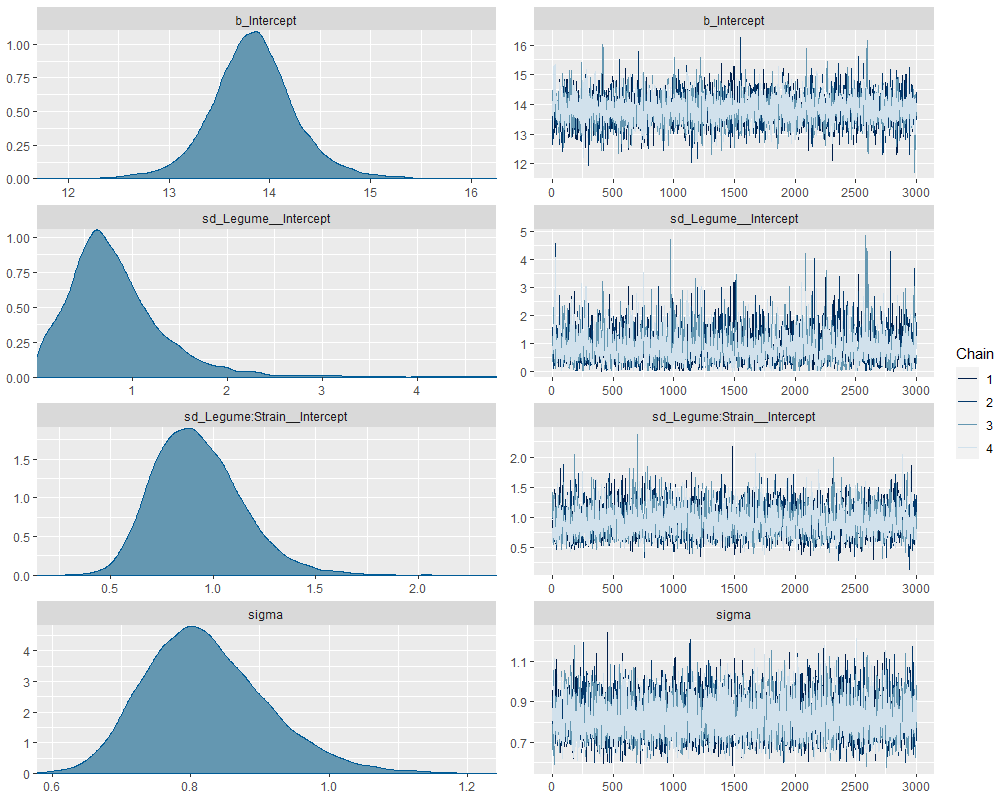


Supplementary material 24- Density plot (left) and trace plot (right) of the Monte Carlo iterations for the model parameters estimated from the data on VOC . The plot shows four lines with different colours, indicating four independent runs of the algorithm. The uniform spread of the iterations and the lack of a clear trend in the time series is a support for the convergence of the algorithm.

Supplementary material 25- GC

| **Strain** | **Soybeans** | **Lupins** | **Chickpeas** | **Red lentils** | **Brown beans** | **Green peas** |
| --- | --- | --- | --- | --- | --- | --- |
| **Bs 1.8** | 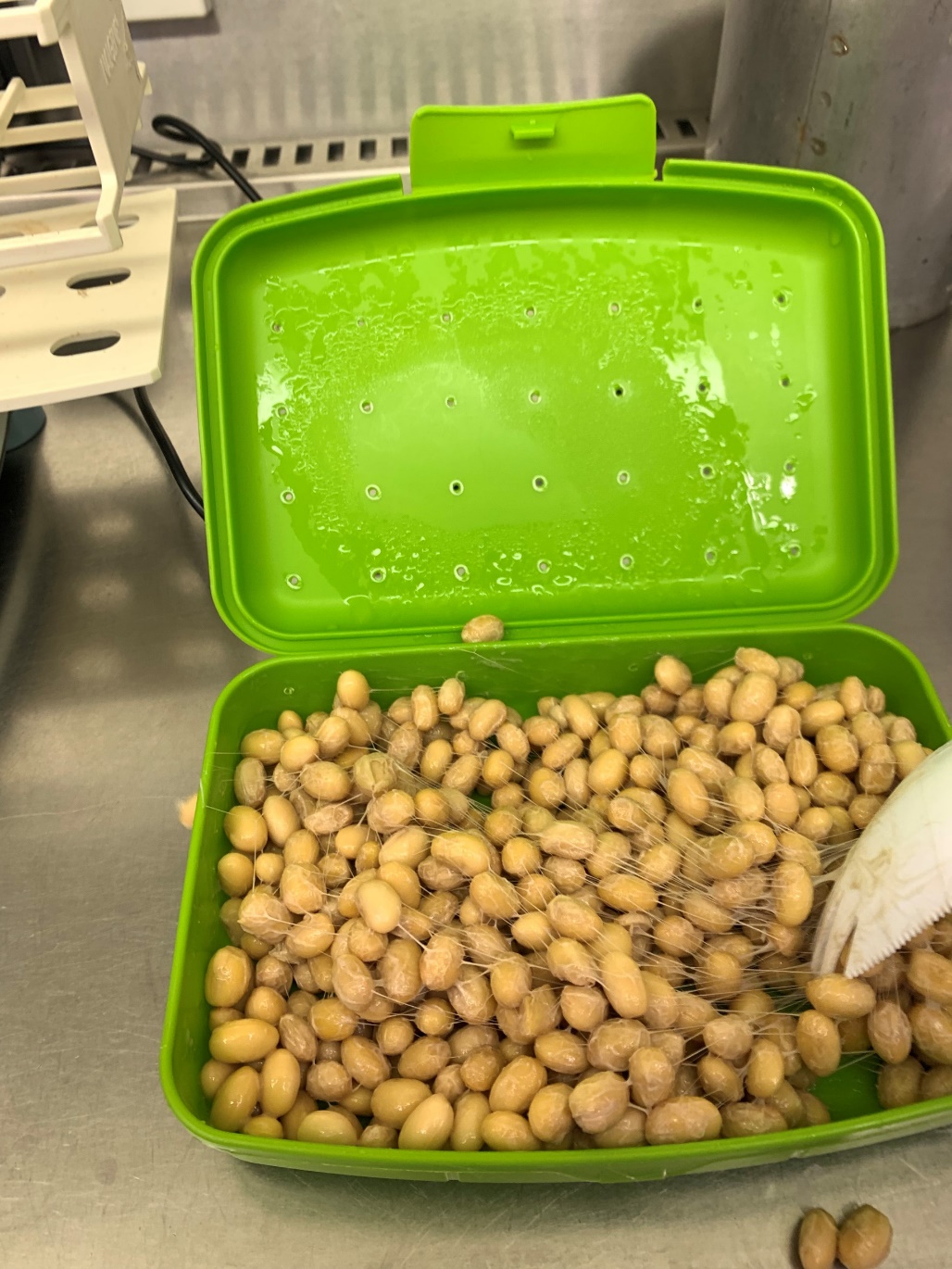 | 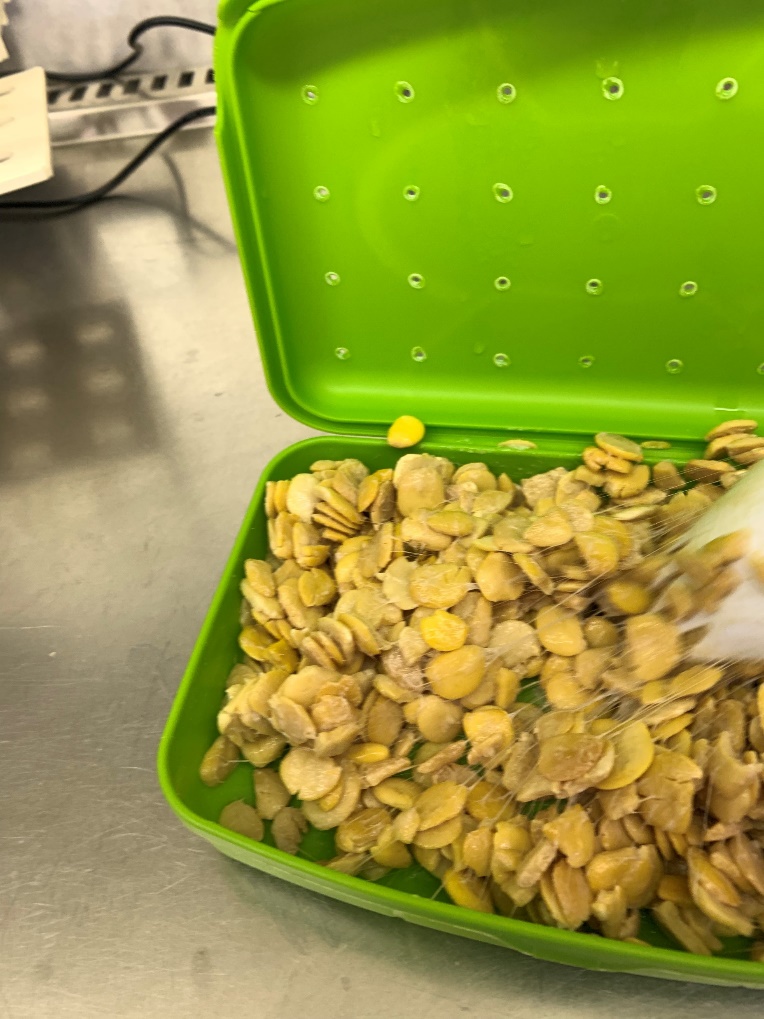 | 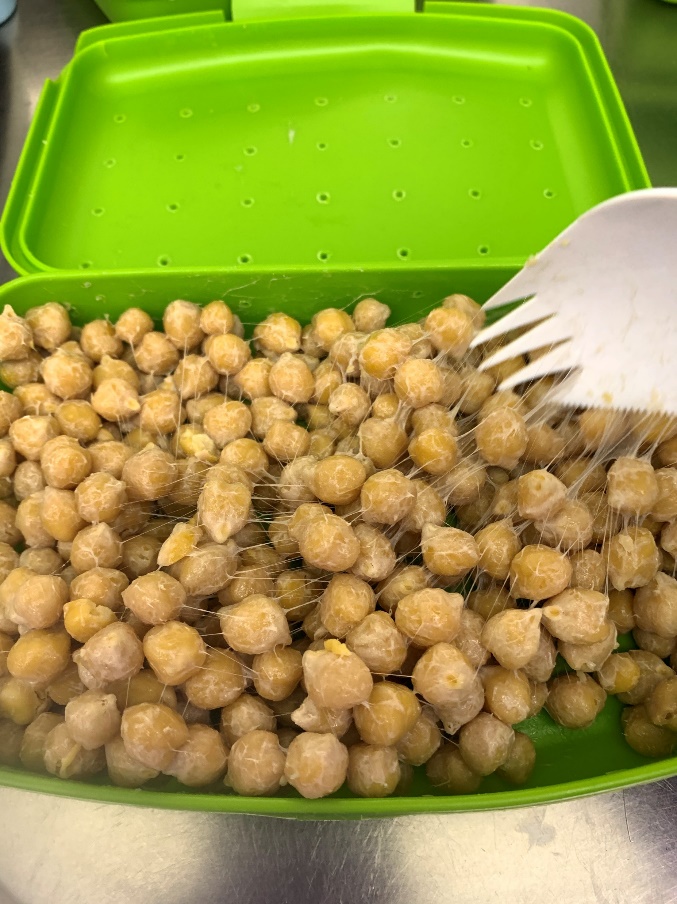 | 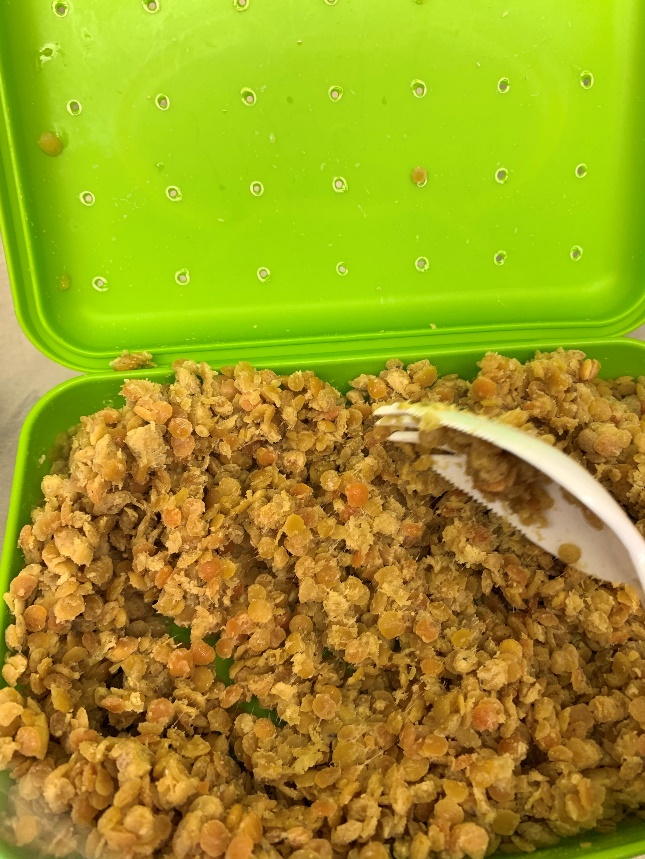 | 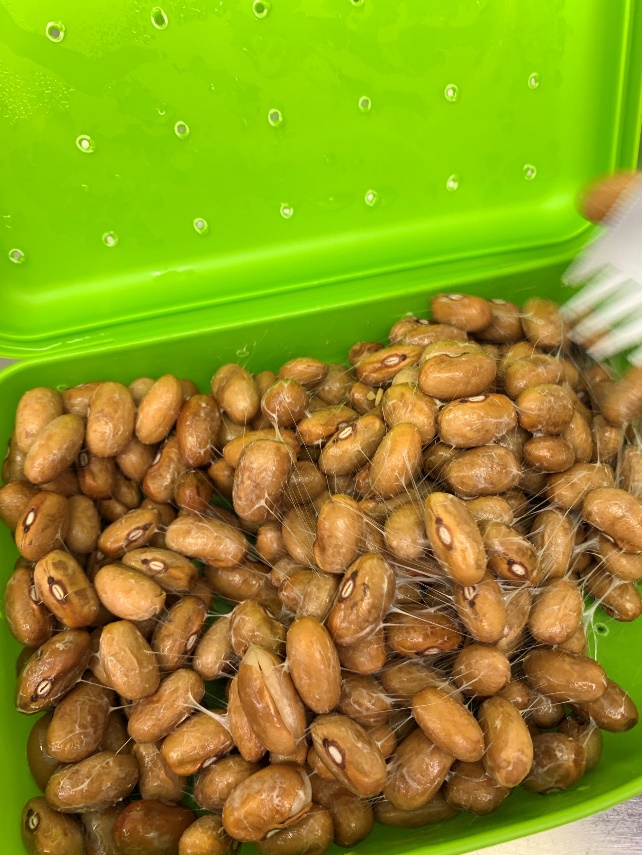 | 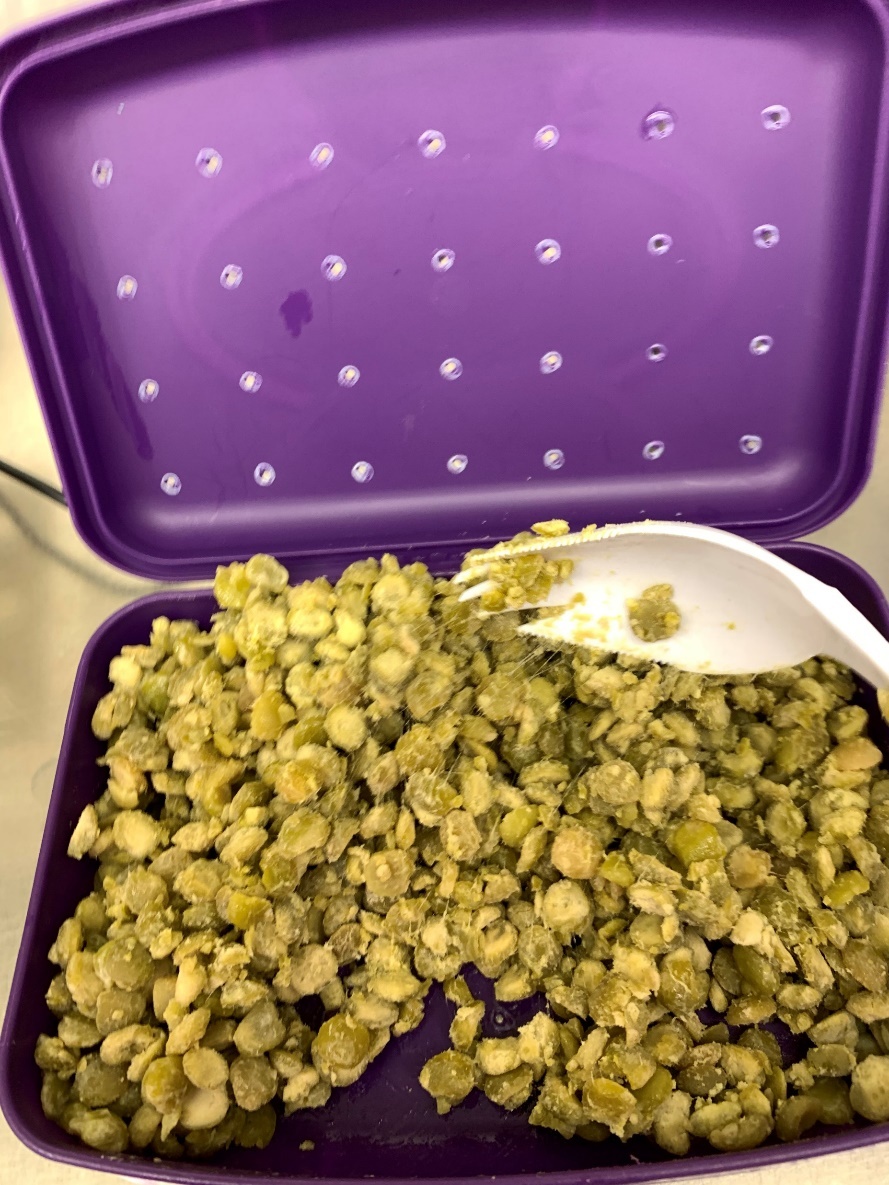 |
| **Bs 2.8** | 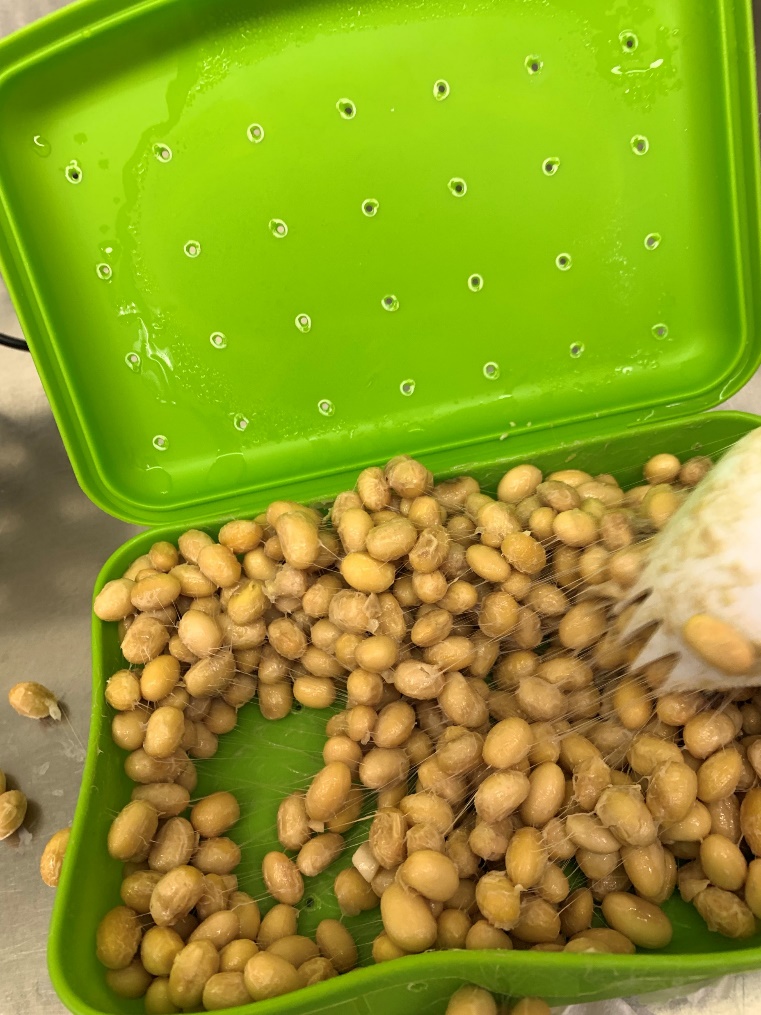 | 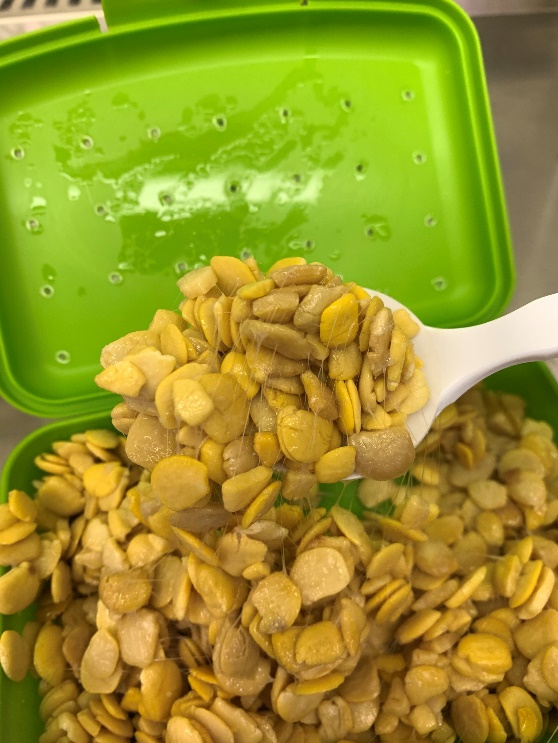 | 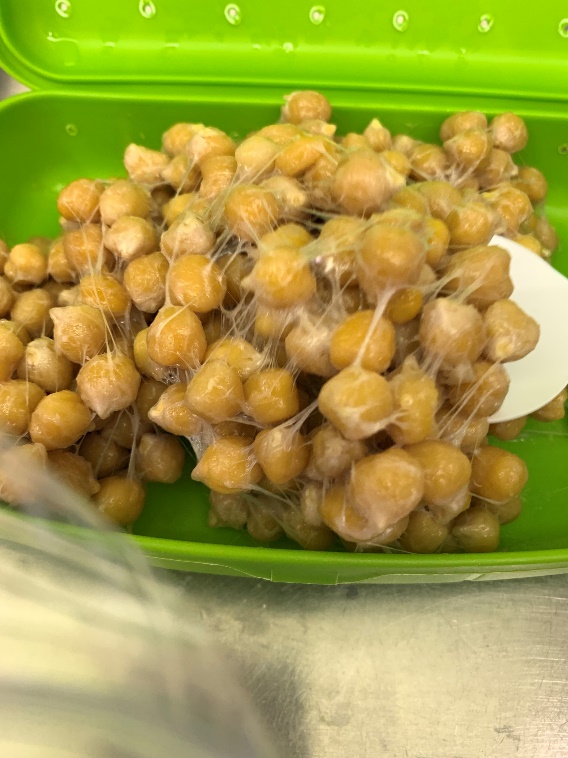 | 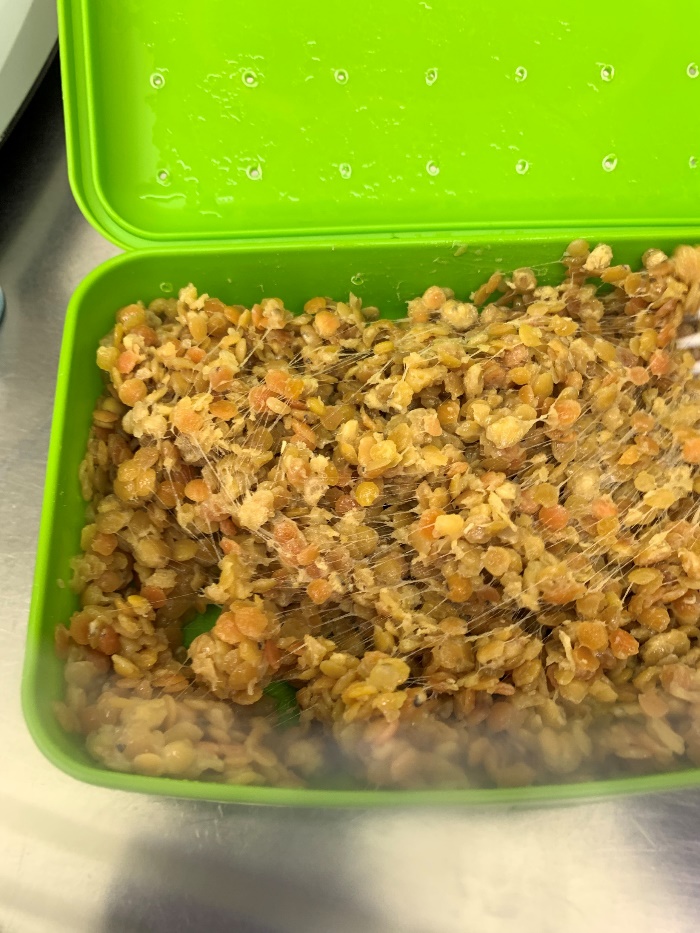 | 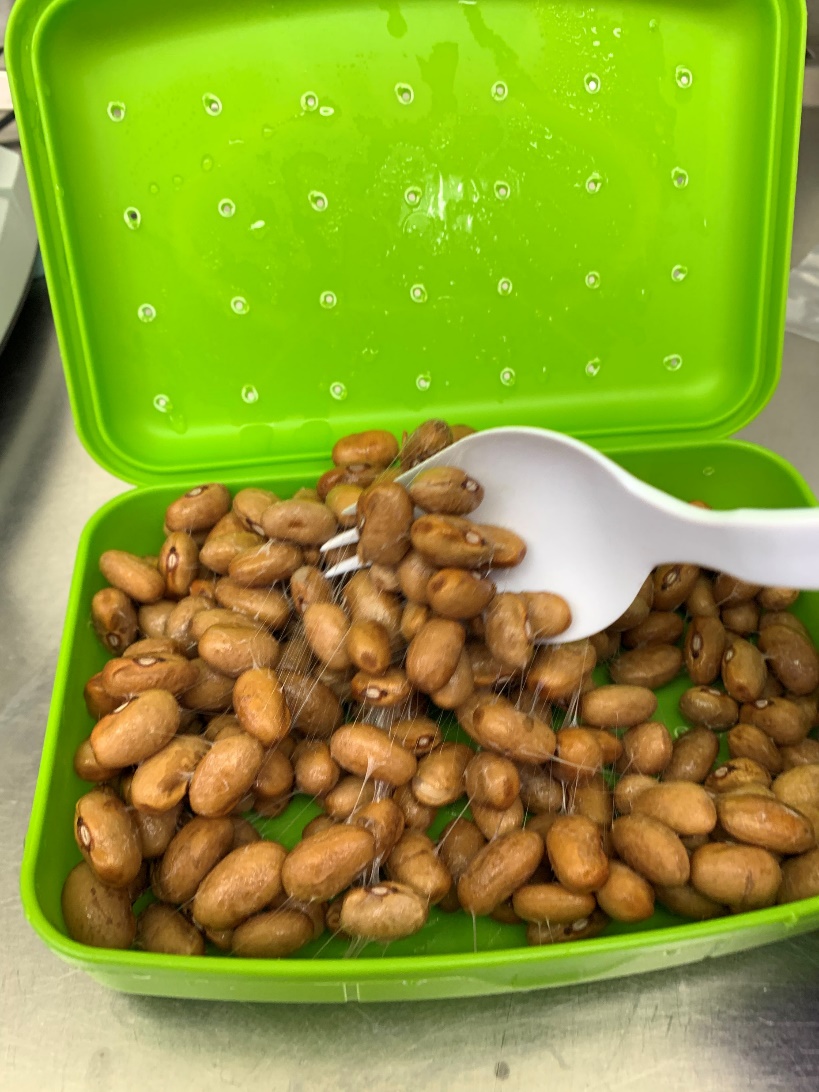 | 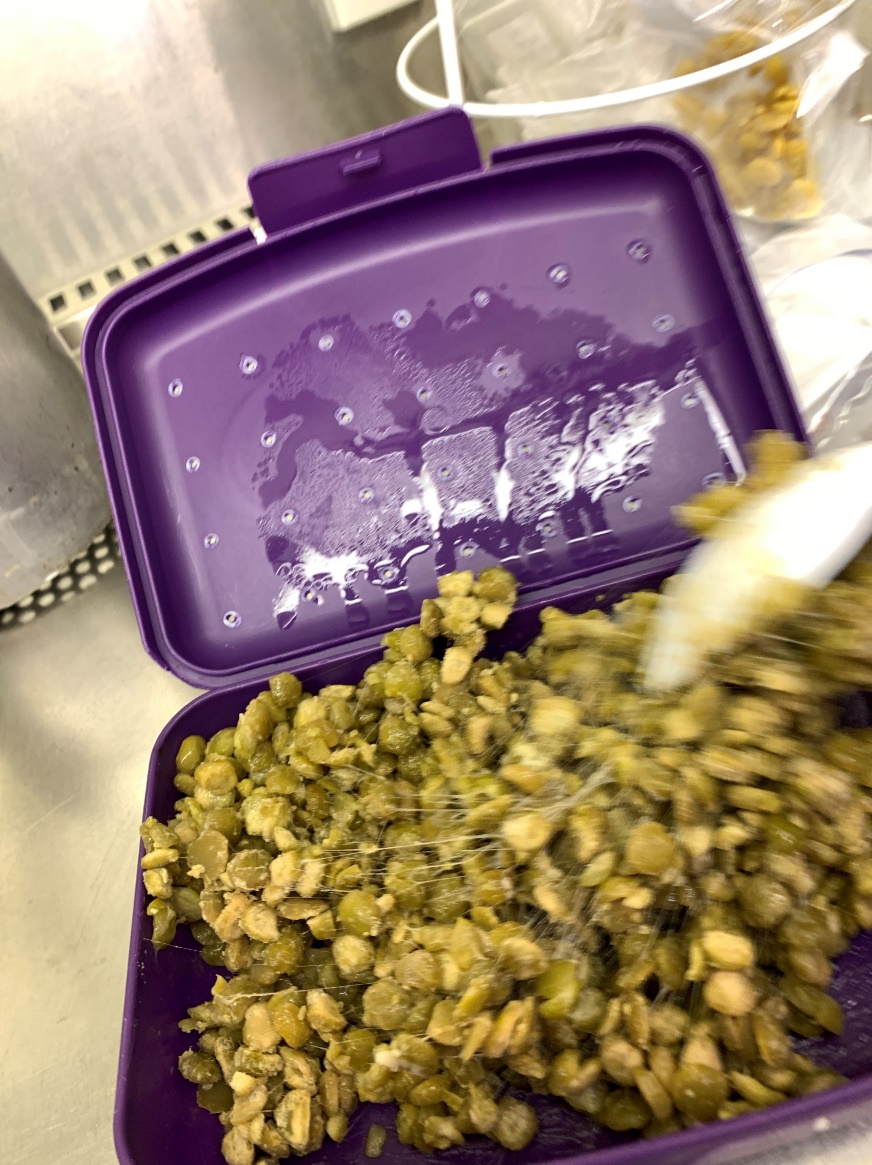 |
| **Bs 92** | 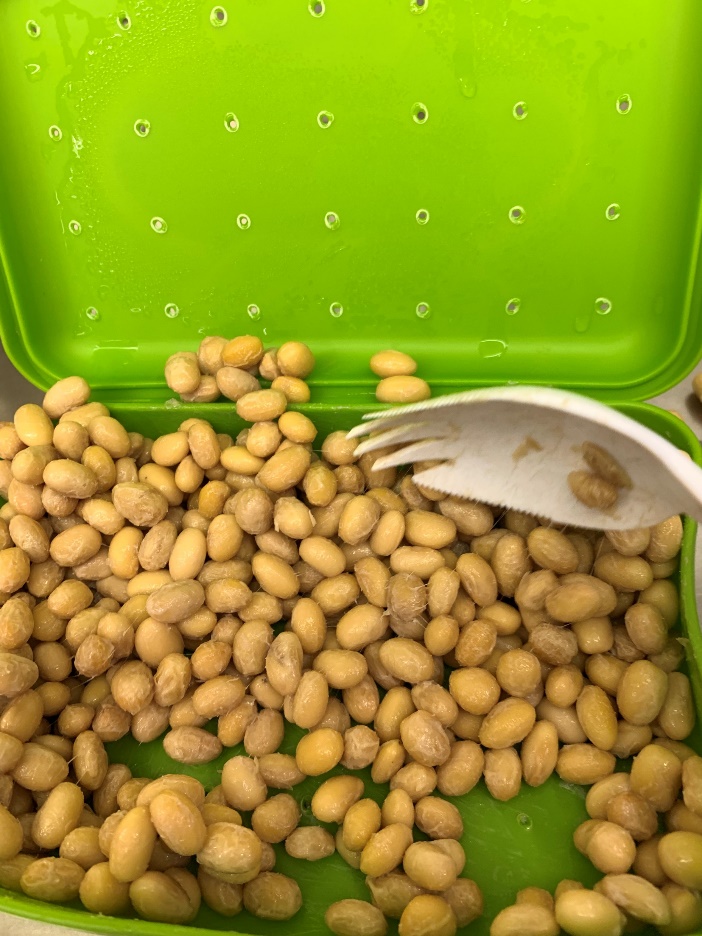 | 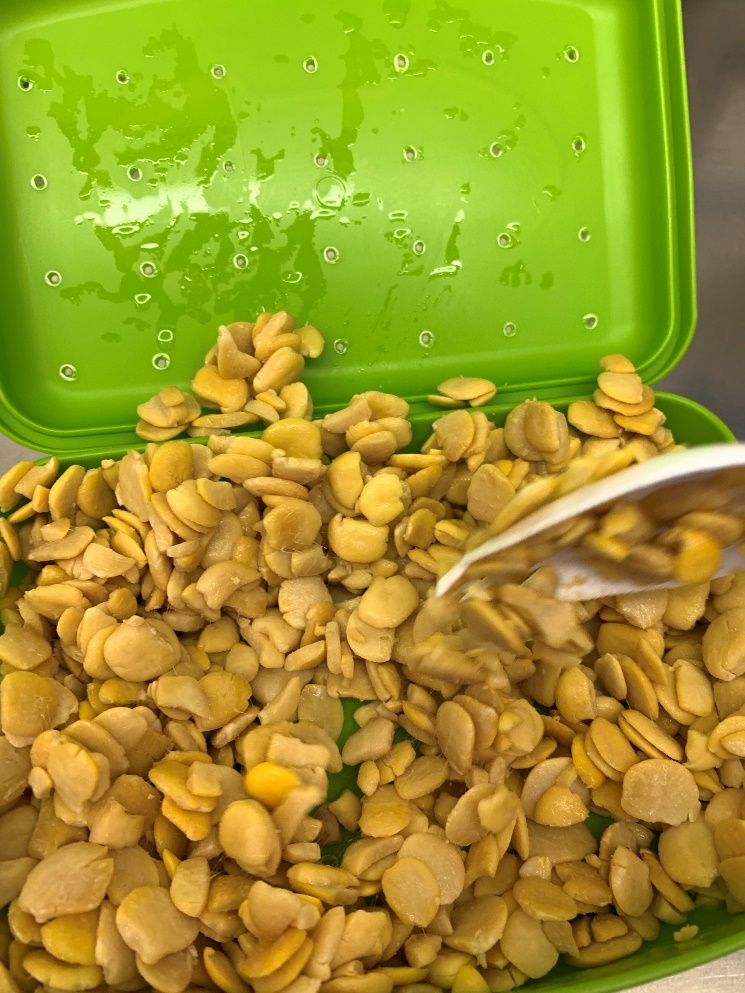 | 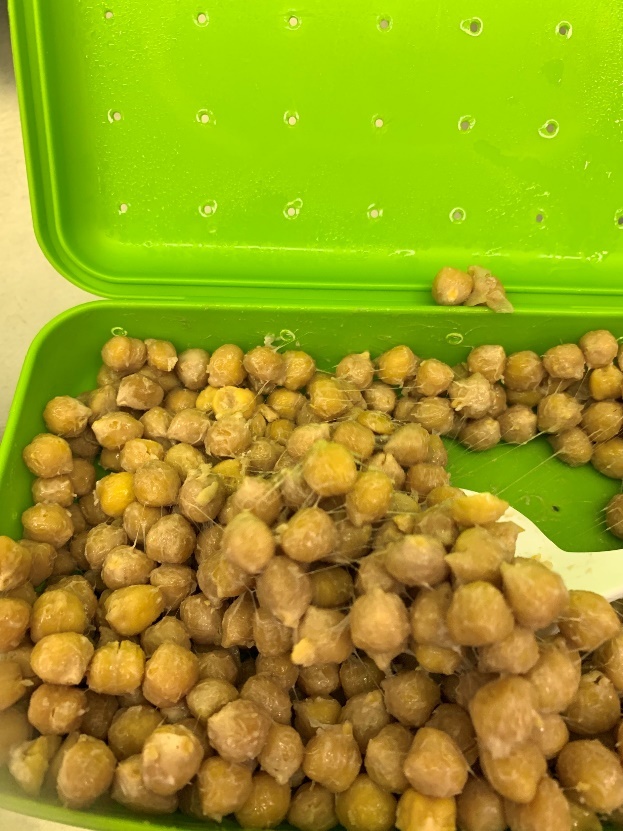 | 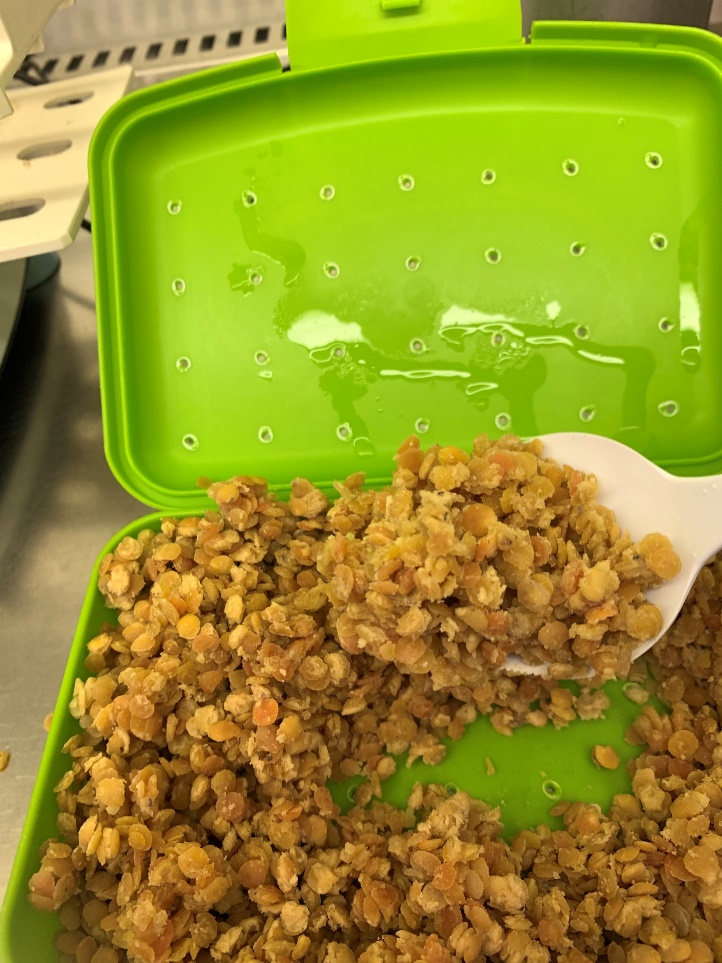 | 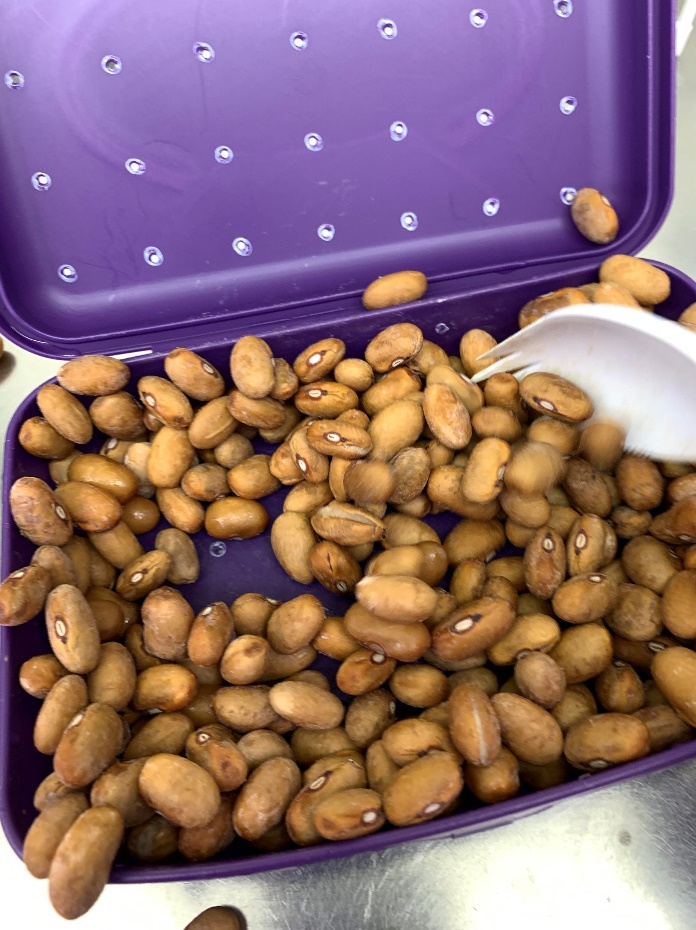 | 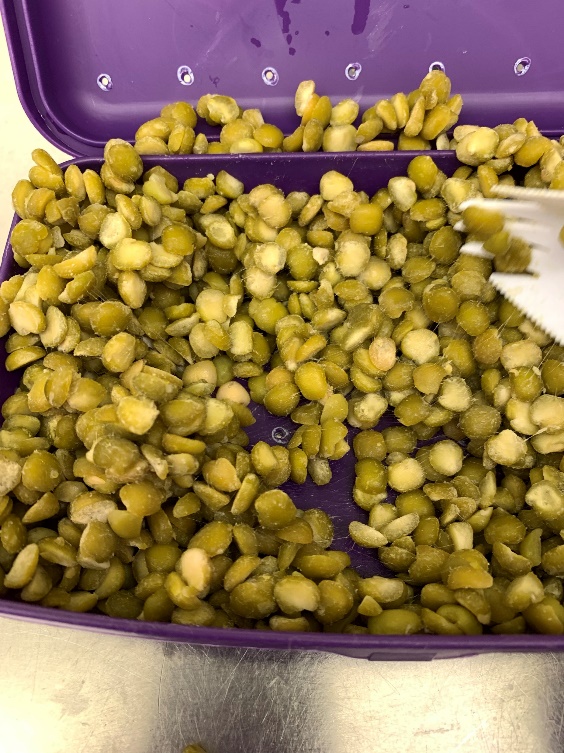 |

Supplementary material 25- Pictures of natto taken directly after fermentation and maturation.
